# Fusing α and β subunits of the fungal fatty acid synthase leads to improved production of fatty acids

**DOI:** 10.1038/s41598-020-66629-y

**Published:** 2020-06-17

**Authors:** Florian Wernig, Sandra Born, Eckhard Boles, Martin Grininger, Mislav Oreb

**Affiliations:** 1 Institute of Molecular Biosciences, Faculty of Biological Sciences, Goethe University Frankfurt, Frankfurt am Main, Germany; 2 Institute of Organic Chemistry and Chemical Biology, Buchmann Institute of Molecular Life Sciences, Goethe University Frankfurt, Frankfurt am Main, Germany

**Keywords:** Metabolic engineering, Enzyme mechanisms

## Abstract

Most fungal fatty acid synthases assemble from two multidomain subunits, α and β, into a heterododecameric FAS complex. It has been recently shown that the complex assembly occurs in a cotranslational manner and is initiated by an interaction between the termini of α and β subunits. This initial engagement of subunits may be the rate-limiting phase of the assembly and subject to cellular regulation. Therefore, we hypothesized that bypassing this step by genetically fusing the subunits could be beneficial for biotechnological production of fatty acids. To test the concept, we expressed fused FAS subunits engineered for production of octanoic acid in *Saccharomyces cerevisiae*. Collectively, our data indicate that FAS activity is a limiting factor of fatty acid production and that FAS fusion proteins show a superior performance compared to their split counterparts. This strategy is likely a generalizable approach to optimize the production of fatty acids and derived compounds in microbial chassis organisms.

## Introduction

Fungal fatty acid synthases (FAS) are prototypical multi-domain molecular machines that form a barrel-like structure. Acyl carrier protein (ACP) domains, spanning their reaction chambers and shuttling substrates and intermediates between the individual catalytic domains, facilitate compartmentalized FA synthesis^1,2^.

Genome sequence analyses has characterized fungal FAS as heterogeneous family comprising currently six gene-topological variations. Most fungal species encode FAS subunits as two multi-domain polypeptides, α and β, that assemble to a hetero-dodecamer (α_6_β_6_). In an elegant study, Bukhari *et al*.^3^ have shown that fungal multifunctional FAS have evolved from monofunctional enzymes that were fused to form a single-gene encoded enzyme as an evolutionary intermediate. Intriguingly, at a later point, gene splitting at various (species specific) positions led to a set of two-genes encoded fungal FAS^3–6^. As an evolutionary late event, gene splitting was non-invasive to the overall structure, and also did not affect the assembly pathway, which was already established before on the single-gene variant^3,6^. This view is in line with recent findings proposing that protein complexes are under strong evolutionary selection for ordered assembly pathways^7^.

Recently, Shiber *et al*.^8^ revealed that yeast FAS assembly is initiated via the cotranslational interaction of the subunits α and β. It was shown that the N-terminus of the α-subunit (encoded by *FAS2*) is engaged by the β-subunit (*FAS1*) for cotranslational substructure folding. As substantiated in a further study on the molecular basis of cotranslational assembly, the C-terminus of β and the N-terminus of α undergo specific interactions while forming the MPT domain. Cotranslational assembly of yeast FAS is not restricted to the naturally occurring splitting site, but the protein can also assemble when subunit borders are shifted. Further, yeast FAS assembles when subunits are fused to a single polypeptide^9^.

Here, we investigated whether these recent findings can be translated into biotechnological application. Given that many industrially relevant compounds are based on fatty acids (FA) and their derivatives (which are currently extracted from plants or synthesized from petrochemicals), engineering microbial cells for production of FA has recently become one of the major targets in biotechnology. Whereas a plethora of strategies to improve the supply of precursor molecules (acetyl-CoA and malonyl-CoA) or redox-cofactors (NADPH) for FA biosynthesis was developed and led to considerable successes as previously reviewed^10,11^, only few studies focused on engineering of FAS enzymes in *S. cerevisiae*, mainly with the aim to control the chain length of produced FA^12–14^. Since upstream pathway engineering can unfold its full potential only if FAS has sufficient capacity to process the precursor molecules, FAS genes are usually overexpressed, e.g. by using strong promoters and/or plasmids^12,15,16^. This can lead to a disbalanced synthesis of FAS α and β subunits in engineered cells and, as a consequence, to degradation of superfluous subunits in the proteasome to maintain the stoichiometry of the complex^17^. We reasoned that fusing α and β subunits in one polypeptide chain^9^ could be beneficial to avoid such undesired side-effects of a deregulated expression and promote cotranslational assembly of the FAS complex. To test the concept with a reliable readout, we used a FAS variant previously developed^12,18^ for production of octanoic acid (OA), a C8 FA that is secreted out of the cells and readily detected in culture supernatants. We show that the single-polypetide FAS is superior to the split-subunit version. The underlying principle likely represents a generically aplicable strategy to increase type I FAS-based production of FA and derived chemicals.

## Results and Discussion

### Construction and functionality of fused FAS subunit**s**

**Figure 1:**
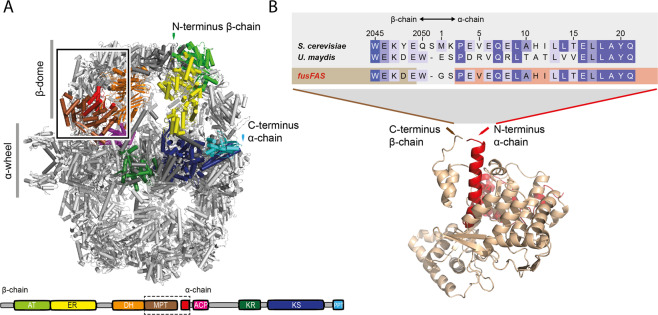
Structure of yeast FAS and the α/β interface. (**A**) Cartoon representation of the X-ray crystallographic structure of *S. cerevisiae* FAS (PDB-code: 3hmj)^28^ with one β subunit and one α subunit shown in color code of the functional domains as schematically depicted below. The MPT fold is comprised of both subunits (β part in brown and α part in red). The region shown in more detail in (**B**) is framed in the structure. Nomenclature: acetyl transferase (AT), enoyl reductase (ER), dehydratase (DH), malonyl-palmitoyl-transferase (MPT), acyl carrier protein (ACP), ketoacyl reductase (KR), ketoacyl synthase (KS) and phosphopantetheine transferase domain (PPT). (B) Structure of the MPT domain of *S. cerevisiae* FAS in cartoon representation and color coded as in (A). In fusFAS, chains are linked by a short sequence derived from the single-chain *Ustilago maydis* FAS. An alignment of the relevant sequence regions is shown.

To synthesize both *S. cerevisiae* FAS subunits as a single polypeptide (“fusFAS”), *FAS1* (encoding the β subunit) and *FAS2* (α subunit) open reading frames (ORFs) were connected by a sequence encoding a linker derived from the single-chain *Ustilago maydis* FAS^9^ (Fig. 1).

**Figure 2:**
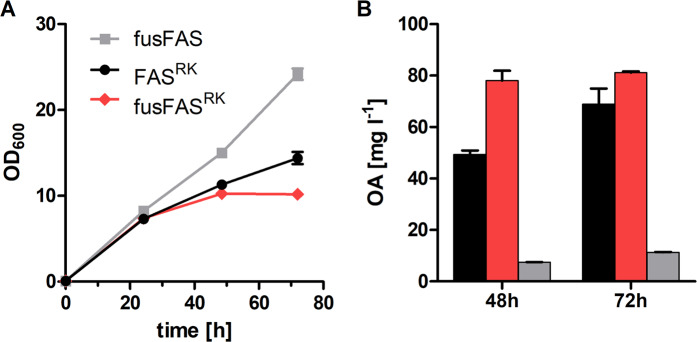
Functionality of FAS fusion constructs. The FAS variants FAS^RK^, fusFAS^RK^ and fusFAS were expressed in the strain SHY34 (*Δfas1 Δfas2 Δfaa2*) cultivated in buffered YPD medium. The growth was assessed by measuring OD_600_ over time (**A**). Octanoic acid titers in culture supernatants were determined by gas chromatography after 48 and 72 h of cultivation (**B**). The same color code is used in both panels. Mean values and standard deviations of biological duplicates are shown. Error bars may be smaller than the symbols.

The fused ORFs were placed under the control of the *FAS1* promoter and *FAS2* terminator and inserted into centromeric plasmids. As a control, we used a plasmid containing *FAS1* and *FAS2* as separate ORFs flanked by their native promoters and terminators (split FAS). First, we compared the ability of these constructs to complement the growth defect of the FAS deficient strain SHY34 in FA-free media. Both plasmids conferred the same growth rate (Supplementary Fig. S3), demonstrating that the fusion strategy does not negatively affect the FAS function. For production of OA, we introduced the R1834K substitution within the Fas1 chain, which was previously shown to promote the production of short and medium chain FA^12,18^ into the fusion construct (fusFAS^RK^) and into the split FAS plasmid (FAS^RK^). In accordance with previous observations^12^, the mutated constructs conferred slower growth rates compared to the wildtype fusFAS (Fig. 2A), due to their reduced ability to synthesize the essential long chain (C16 and C18) FA^12^ and cytotoxicity of the produced OA^19^.

Next, we compared OA titers produced by SHY34 expressing different FAS variants in shake flask fermentations (Fig. 2B). In addition to the deletion of genomic *FAS1* and *FAS2* copies, *FAA2* gene encoding the medium chain fatty acyl-CoA synthetase was deleted in this strain to minimize degradation of octanoic acid via β-oxidation as described previously^15,20^. fusFAS^RK^ expression resulted in a higher accumulation of extracellular OA compared to the two-gene-encoded FAS^RK^ at both time points. Strikingly, with fusFAS^RK^ the difference was more pronounced at the earlier time point (corresponding to an increase of 58% compared to the split enzyme) indicating that a higher productivity (defined as product formation per time) can be achieved by the fusion of subunits. Moreover, the production of two byproducts with different chain lengths, hexanoic acid and decanoic acid, was also increased with the fusion construct (Supplementary Fig. S4), suggesting that the approach is generalizable and not restricted to the production of OA.

Based on these data, it may be hypothesized that the assembly of the FAS complex occurs faster, as anticipated. Moreover, the equimolar stoichiometry of both subunits in the fusion protein can indirectly have a positive effect on cellular physiology by obviating the energetically wasteful cycles of synthesis and degradation of superfluous subunits^17^, which is likely to occur if two genes are separately overexpressed with strong constitutive promoters. Although we cannot rule out that the FAS complex assembled from fused subunits is more resistant to proteolytic degradation, this hypothesis contradicts our observations, since autophagy of FAS is initiated during starvation^21^ (i.e. at later stages of cultivation), where the benefit of fusFAS^RK^ expression was less pronounced (see Fig. 2B at 72 h).

### Improving the expression of engineered FAS fusion constructs

**Figure 3:**
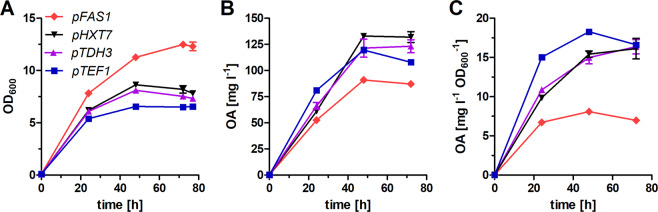
Expression of fusFAS with different promotors. fusFAS^RK^ was expressed from strong promotors (*pTEF1*, *pTDH3*, *pHXT7*^1–3^^29^) or *pFAS1* in strain SHY34 (*Δfas1 Δfas2 Δfaa2*). Comparison of growth (**A**) and OA production (**B**) in buffered YPD medium over a period of 72 h is shown. In (**C**), the titers were normalized to the OD_600_ of the respective culture. Mean values and standard deviations of biological duplicates are shown. Error bars may be smaller than the symbols.

The results presented above indicate that FAS activity is at least one of the limiting factors for OA production. Increased transcription and translation efficiency could therefore lead to further improvements of the production rate. We first performed a codon-optimization of the FAS sequences (for details see SI), but this had only a marginal, if any, effect on OA titers (see Supplementary Fig. S5). We next sought to improve the transcriptional control of FAS constructs. For this, well-known and extensively characterized strong constitutive promoters *pHXT7*^*−1—*392^, *pTDH3* and *pTEF1*^22,23^ were selected. We expressed the fusFAS^RK^ under the control of these three promotors or *pFAS1* as a reference in SHY34 and compared the growth (Fig. 3A) and OA production (Fig. 3B) of the transformants.

Again, with all fusFAS^RK^ constructs the maximum titer was reached after 48 h hours independently of the promoter. The plasmid with the truncated *pHXT7* led to the highest titer after 48 h (133.00 ± 0.6 mg l^*−*1^) and 72 h (131.9 ± 3.6 mg l^*−*1^) of fermentation, an increase of 50% compared to the native *pFAS1* (87.1 ± 1.4 mg l^*−*1^) after 72 h. Interestingly, the highest titers at 24 h were reached with the *pTEF1* construct, which correlated with decreased cell growth of the corresponding strain, likely due to OA toxicity. To take into account the trade-off between the biomass and OA yields, we calculated the specific OA titers (mg l^*−*1^ OD_600_^*−*1^, Fig. 3C). This analysis shows that, if cell proliferation is not desired (e.g. in high cell density fermentations), *pTEF1* is the promoter of choice, whereas *pHXT7* (or *pTDH3*) can be preferably used for a low inoculum culture.

### Co-expression of WT and engineered FAS variants

**Figure 4:**
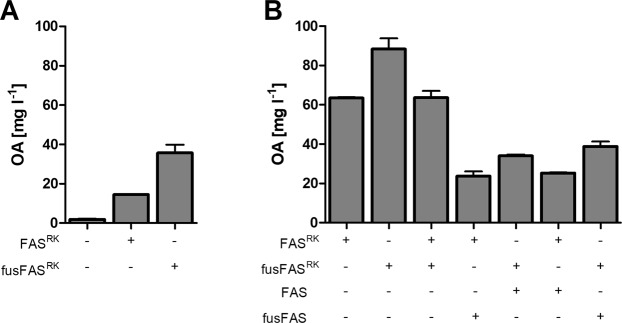
Co-expression of engineered and WT-FAS variants. (**A**) FAS^RK^ and fusFAS^RK^ were expressed from plasmids where indicated (“+”) in the strain SHY24 (*Δfaa2*) that additionally has native *FAS* alleles in the genome. As a control, the cells transformed with the empty vector were used (left column). In (**B**), the indicated combinations of FAS plasmids were expressed in the FAS-deficient strain SHY34 (*Δfas1 Δfas2 Δfaa2*). The cells containing only one FAS plasmid (in the first two columns) were additionally transformed with appropriate empty vectors. Strains were cultivated in YPD with potassium phosphate buffer. For plasmid maintenance hygromycin (100 mg l^*−*1^) (A) or hygromycin (100 mg l^*−*1^) plus G418 (200 mg l^*−*1^) (B) were used. Mean values and standard deviation of two biological replicates at 72 h of fermentation are shown.

In our previous work, the mutated FAS^RK^ variant was expressed in FAS deficient (*Δfas1 Δfas2*) strains to unambiguously characterize the properties of the engineered enzyme. As observed before^12^ with FAS^RK^ and confirmed in Fig. 2A for fusFAS^RK^, the mutated enzyme does partially complement the requirement of the strain for C16 and C18 FA due to its leaky chain length control, but there is a significant growth defect correlating with the production of OA (see Fig. 3). Since slow growth is an undesired trait from a biotechnological viewpoint, we wondered whether the mutated enzymes could be expressed in a FAS WT background to produce OA in a normally proliferating strain. An obvious pitfall of simultaneously expressing different variants of the same FAS subunits is the possible formation of heterogeneous complexes (i.e. assembling Fas1^R1834K^ and Fas1^WT^ β chains in the same α_6_β_6_ dodecamer). We hypothesized that the concomitant expression of fusFAS^RK^ and (split) WT FAS would favor two homogenous FAS entities, as the topology of the fusion construct (aminoterminus-β-α-carboxyterminus) would not allow for the interaction with the termini of the split subunits (which engage via an interaction of the C-terminus of β with the N-terminus of the nascent α-chain)^8,9^. Hence, we expressed fusFAS^RK^ or FAS^RK^ in SHY24, which has WT *FAS1* and *FAS2* alleles in the genome and measured OA titers in culture supernatants (Fig. 4A).

In line with results shown in Fig. 2B, the fused enzyme exhibited superior performance, but the titers were overall lower compared to the FAS deficient strain (compare Figs. 2B and 4A). To rule out any unspecific effects of the strain background, we transformed plasmids with fused and split FAS variants with or without the R1834K mutation in different combinations into the FAS deficient strain SHY34. In accordance with other results presented here, fusFAS^RK^ showed higher productivity than FAS^RK^ and the presence of WT FAS reduced the OA titers in all combinations tested (Fig. 4B). The latter observation could be hypothetically explained by different mechanisms (including combinations thereof): (i) competition for substrates and cofactors (acetyl-CoA, malonyl-CoA, NADPH) between mutated and WT FAS; (ii) elongation of octanoyl-CoA released from mutated FAS by WT FAS as observed *in vitro* (Pirson *et al*., 1973) and (iii), as outlined above, formation of heterogeneous complexes, in which the WT subunits could elongate octanoyl-CoA released by mutated ones within the same FAS reaction chamber. However, the surprising finding that the co-expression of FAS^RK^ and fusFAS^RK^ yields less OA than fusFAS^RK^ alone cannot be explained by hypotheses (i) and (ii) and suggests that an interaction, including the formation of a heterogeneous complex, may occur between fused and singular subunits at some stage of complex assembly. In such a scenario, the physical interaction between fused and split subunits could have a negative kinetic effect on the assembly of the FAS complex and consequently lead to lower OA titers. However, other hypotheses to explain the observed effect cannot be ruled out at present. Regardless of the underlying mechanism, our data indicate that the interferences between engineered and WT FAS activities cannot be circumvented by expressing the fusion proteins.

## Conclusion

Taken together, our data demonstrate that fusing α and β subunits of FAS in one polypeptide chain leads to a substantially higher FAS activity, measured as increased production of OA. Although the elucidation of the underlying mechanism is not in the scope of this study, it is - based on previously published research - reasonable to assume that an increased assembly rate and balanced stoichiometry of the subunits may be responsible for the observed effect. To optimize the expression of the fused FAS constructs, we identified a set of suitable promoters. Importantly, we further show that the simultaneous presence of WT and engineered FAS variants decreases the OA titers by physical and/or metabolic crosstalk of different enzyme populations, which cannot be circumvented by expressing a single-chain version of the engineered FAS. The principles described here very likely apply to the biotechnological production of any FAS-derived molecules. Moreover, fusing the subunits of cotranslationally assembled protein complexes may be a generically applicable strategy, reaching beyond the production of FA.

## Materials and Methods

### Strain construction and transformation

Yeast strains used in this study are listed in Table 1. The strain SHY24 was constructed by deleting the *FAA2* locus in BY4741 using the plasmid pRCC-N-faa2 (see Supplementary Table S1) by CRISPR-Cas9 meditated gene deletion as described previously^24^. For this, a donor DNA together with the CRISPR-Cas9 plasmid encoding for the Cas9 and the guide RNA with a protospacer sequence targeting specifically *FAA2* (GAAGATTTTGAAACCTTACG) was transformed into yeast cells. The strain SHY34 resulted from the previously described strain RPY21^15^ by deletion of two *kanMX4* markers which were present in the RPY21 genome as remnants of *FAS1* and *FAS2* deletion by the same CRISPR-procedure (pRCC-N-kanMX4; protospacer sequence: TTACTCACCACTGCGATCCC). RPY21 has a BY background and is based on strain BY.PK1238_1A_KO^12^, in which *FAA2* was previously deleted^15^ as described above for SHY24.

**Table 1:** Yeast strains used in this study.

**Strain name**	**Relevant genotype**	**Reference/Source**
BY4741	*MATa his3Δ1 leu2Δ0 met15Δ0 ura3Δ0*	(Brachmann *et al*., 1998)^29^
SHY24	*MATa his3Δ1 leu2Δ0 met15Δ0 ura3Δ0 ∆faa2*	This work
SHY34	*MATα ura3Δ0 his3Δ0 leu2Δ0 TRP1 lys2Δ0 MET15 ∆fas1 ∆fas2 ∆faa2*	This work

Transformations were performed following the frozen competent cell protocol^25^, whereas SHY34 was transformed by a slightly modified method previously described^12^. Specifically, because strain SHY34 is FAS deficient, the cells were cultivated in YPD medium supplemented with oleic acid (2% (w/v) peptone, 1% (w/v) yeast extract, 2% (w/v) glucose, 1.42% (v/v) Tergitol^TM^ solution NP-40, 0.016% (v/v) oleic acid) before transformation with the appropriate plasmid coding for FAS. Transformed yeasts were plated on solid YPD (2% (w/v) peptone, 1% (w/v) yeast extract, 2% (w/v) glucose) containing appropriate antibiotics hygromycin (100 mg l^*−*1^) or G418 (200 mg l^*−*1^) for plasmid selection and grown at 30 °C for two to four days.

### Plasmid construction

Nucleotide sequences of FAS variants used in this study are shown in Supplementary Information and plasmids are listed in Supplementary Table S1. Plasmids were constructed via homologous recombination in yeast^26^. Plasmid fragments were amplified by PCR using oligonucleotides listed in Supplementary Table S2. The assembled plasmids were propagated in and extracted from *E. coli* DH10B by standard procedures.

For replacement of auxotrophy markers by dominant markers, *hphNT1* or *kanMX4* cassettes were amplified from pRS62-H or pRS62-K, respectively, and inserted into the EcoRV cut site of *LEU2* in pRS315 or the MscI cut site of *HIS3* in pRS313 based plasmids.

### Media and cultivation

*Saccharomyces cerevisiae* liquid cultures were grown in shake flasks at 30 °C and 180 rpm in YPD medium as described previously^12^ without supplementation of free FA or with supplementation of oleic acid (0.5 mM and 1% (v/v) Tergitol NP-40 solution Sigma Aldrich, Germany) for the FAS deficient strain. For maintaining plasmids with *hphNT1* or *kanMX4* marker appropriate antibiotics hygromycin (100 mg l^*−*1^) or G418 (200 mg l^*−*1^) were used. The medium was additionally buffered with 100 mM potassium phosphate and adjusted to a pH of 6.5. Main cultures of 50 mL were inoculated from pre cultures to an OD_600_ of 0.1 and grown for 72 h at 30 °C with shaking (200 rpm). Samples for compound extraction were taken at given time points.

### Compound extraction and derivatization

Extraction of free fatty acids in the culture medium was performed as described before^15^. Cells were separated from the medium by centrifugation (3,500 rcf, 10 min) and 10 ml of culture supernatant was mixed with an internal standard (0.2 mg heptanoic acid), 1 mL of 1 M HCl and 2.5 ml of methanol:chloroform (1:1) solution. After phase separation (3,000 rcf, 5 min) the organic phase layer was taken and evaporated in a vacuum concentrator (Concentrator 5301, Eppendorf, Germany). Fatty acids were methylated for GC analysis as described^27^. The extract was dissolved in 200 μL toluene, mixed with 1.5 mL of methanol and 300 μL of 8.0% (w/v) HCl solution and incubated at 100 °C for 3 h to form fatty acid methyl esters (FAME). FAMEs were extracted from the mixture by addition of 1 ml H_2_O and 1 ml hexane. The organic phase was taken for gas chromatography analysis.

### Gas chromatography

The gas chromatography analysis was performed on a Perkin Elmer Clarus 400 system (Perkin Elmer, Germany) equipped with an Elite-5MS capillary column (Ø 0.25 mm; length 30 m; film thickness 1.00 µm) and a flame ionization detector (Perkin Elmer, Germany). 1 μL of sample was analyzed after split injection (1:10) and helium was used as carrier gas (90 kPa). For FAME quantification, the temperatures of the injector and detector were set to 200 and 250 °C, respectively. The following temperature program was applied: run time 42.67 min, start at 50 °C and hold for 5 min; ramp at 10 °C min to 120 °C and hold for 5 min, ramp at 15 °C to 220 °C and hold for 10 min, ramp at 20 °C to 300 °C and hold for 5 min. FAMEs were identified and quantified by comparison with authentic standard substances.

## Supplementary information

Supplementary Information.

## Data Availability

The authors will make available all data (underlying the described findings) without restriction.

## References

[1] Leibundgut M, Maier T, Jenni S, Ban N (2008). The multienzyme architecture of eukaryotic fatty acid synthases. Curr. Opin. Struct. Biol..

[2] Gipson P (2010). Direct structural insight into the substrate-shuttling mechanism of yeast fatty acid synthase by electron cryomicroscopy. Proc. Natl. Acad. Sci. USA.

[3] Bukhari HST, Jakob RP, Maier T (2014). Evolutionary origins of the multienzyme architecture of giant fungal fatty acid synthase. Structure.

[4] Jenni S (2007). Structure of fungal fatty acid synthase and implications for iterative substrate shuttling. Science.

[5] Grininger M (2014). Perspectives on the evolution, assembly and conformational dynamics of fatty acid synthase type I (FAS I) systems. Curr. Opin. Struct. Biol..

[6] Fischer M (2015). Cryo-EM structure of fatty acid synthase (FAS) from Rhodosporidium toruloides provides insights into the evolutionary development of fungal FAS. Protein Sci..

[7] Marsh JA (2013). Protein complexes are under evolutionary selection to assemble via ordered pathways. Cell.

[8] Shiber A (2018). Cotranslational assembly of protein complexes in eukaryotes revealed by ribosome profiling. Nature.

[9] Fischer M (2020). Analysis of the co-translational assembly of the fungal fatty acid synthase (FAS). Sci. Rep..

[10] Fernandez-Moya, R. & Da Silva, N. A. Engineering Saccharomyces cerevisiae for high-level synthesis of fatty acids and derived products. *FEMS Yeast Res*. **17**; 10.1093/femsyr/fox071 (2017). 10.1093/femsyr/fox071 28961899

[11] Baumann, *et al* Engineering Saccharomyces cerevisiae for production of fatty acids and their derivatives. In The Mycota Vol. II: Genetics and Biotechnology, 3rd edition (In Press), Benz, J. P. & Schipper, K., eds. (Springer, 2020).

[12] Gajewski J, Pavlovic R, Fischer M, Boles E, Grininger M (2017). Engineering fungal de novo fatty acid synthesis for short chain fatty acid production. Nat. Comm..

[13] Zhu Z (2017). Expanding the product portfolio of fungal type I fatty acid synthases. Nat. Chem. Biol..

[14] Zhu Z (2020). Multidimensional engineering of Saccharomyces cerevisiae for efficient synthesis of medium-chain fatty acids. Nat. Catal..

[15] Henritzi S, Fischer M, Grininger M, Oreb M, Boles E (2018). An engineered fatty acid synthase combined with a carboxylic acid reductase enables de novo production of 1-octanol in Saccharomyces cerevisiae. Biotechnol. Biofuels.

[16] Wernig F, Boles E, Oreb M (2020). De novo biosynthesis of 8-hydroxyoctanoic acid via a medium-chain length specific fatty acid synthase and cytochrome P450 in Saccharomyces cerevisiae. Metab. Eng. Comm..

[17] Scazzari M, Amm I, Wolf DH (2015). Quality control of a cytoplasmic protein complex. Chaperone motors and the ubiquitin-proteasome system govern the fate of orphan fatty acid synthase subunit Fas2 of yeast. J. Biol. Chem..

[18] Gajewski J (2017). Engineering fatty acid synthases for directed polyketide production. Nat. Chem. Biol..

[19] Liu P (2013). Membrane stress caused by octanoic acid in Saccharomyces cerevisiae. Appl. Microbiol. Biotechnol..

[20] Leber C, Choi JW, Polson B, Da Silva NA (2015). Disrupted short chain specific β-oxidation and improved synthase expression increase synthesis of short chain fatty acids in Saccharomyces cerevisiae. Biotechnol. Bioeng..

[21] Shpilka T (2015). Fatty acid synthase is preferentially degraded by autophagy upon nitrogen starvation in yeast. Proc. Natl. Acad. Sci. USA.

[22] Hamacher T, Becker J, Gardonyi M, Hahn-Hagerdal B, Boles E (2002). Characterization of the xylose-transporting properties of yeast hexose transporters and their influence on xylose utilization. Microbiology.

[23] Lee ME, DeLoache WC, Cervantes B, Dueber JE (2015). A Highly characterized yeast toolkit for modular, multipart assembly. ACS Synth. Biol..

[24] Generoso WC, Gottardi M, Oreb M, Boles E (2016). Simplified CRISPR-Cas genome editing for Saccharomyces cerevisiae. J. Microbiol. Meth..

[25] Gietz RD, Schiestl RH (2007). Frozen competent yeast cells that can be transformed with high efficiency using the LiAc/SS carrier DNA/PEG method. Nat. Protoc..

[26] Oldenburg KR, Vo KT, Michaelis S, Paddon C (1997). Recombination-mediated PCR-directed plasmid construction *in vivo* in yeast. Nucleic Acids Res..

[27] Ichihara K‘i, Fukubayashi Y (2010). Preparation of fatty acid methyl esters for gas-liquid chromatography. J. Lipid Res..

[28] Johansson P (2008). Inhibition of the fungal fatty acid synthase type I multienzyme complex. Proc. Natl. Acad. Sci. USA.

[29] Brachmann, C. B. *et al*. Designer deletion strains derived from Saccharomyces cerevisiae S288C. A useful set of strains and plasmids for PCR-mediated gene disruption and other applications. *Yeast* **14**, 115–132, 10.1002/(SICI)1097-0061(19980130)14:2<115::AID-YEA204>3.0.CO;2-2 (1998). 10.1002/(SICI)1097-0061(19980130)14:2<115::AID-YEA204>3.0.CO;2-2 9483801

