# Supplementary material for: Fusing α and β subunits of the fungal fatty acid synthase leads to improved production of fatty acids
Source: Sci Rep. 2020 Jun 17;10:9780. doi: 10.1038/s41598-020-66629-y (PMC7300031; doi:10.1038/s41598-020-66629-y)
Supplement: Supplementary file 1 — Supplementary Information. [file 41598_2020_66629_MOESM1_ESM.pdf]

Supplementary Information

Fusing α and β subunits of the fungal fatty acid synthase leads to improved production of fatty acids

Florian Wernig[1], Sandra Born[1], Martin Grininger[2], Eckhard Boles[1] & Mislav Oreb[1*]

[1]Institute of Molecular Biosciences, Faculty of Biological Sciences, Goethe University Frankfurt, Frankfurt am Main, Germany
[2]Institute of Organic Chemistry and Chemical Biology, Buchmann Institute of Molecular Life Sciences, Goethe University Frankfurt, Frankfurt am Main, Germany

*corresponding author:
Dr. Mislav Oreb
Institute of Molecular Biosciences
Max-von-Laue Straße 9
60438 Frankfurt
Germany
Telephone +49 (0)69 798 29331
Telefax +49 (0)69 798 29527
E-Mail m.oreb@bio.uni-frankfurt.de

Supplementary Table S1 | Plasmids used in this study. Under "description" the relevant genetic elements are listed. AmpR, Ampicillin resistance; prefixes "p" and "t" denote promoters and terminators, respectively; MCS, multiple cloning site. Other elements listed follow conventional nomenclature or (for FAS variants) the nomenclature used in the manuscript.

Laboratory stock code	Plasmid name	Description	Used in Figure	Reference
ASB22	pRS62-H	*2μ, AmpR, HphNT1, pHXT7-MCS-tCYC1*	-	Farwick et al., 2014
ASB23	pRS62-K	*2μ, AmpR, kanMX4, pHXT7-MCS-tCYC1*	-	Farwick et al., 2014
SHV61	pRS315-FAS1^{RK}-FAS2	*CEN6/ARS4, AmpR, LEU2, pFAS1-FAS1^{R1834K}-tFAS1, pFAS2-FAS2-tFAS2*	Fig. 2, S4	This study
FWV113	pRS315K-FAS1^{RK}-FAS2	*CEN6/ARS4, AmpR, kanMX4, pFAS1-FAS1^{R1834K}-tFAS1, pFAS2-FAS2-tFAS2*	Fig. 4a,b; S5	This study
FWV124	pRS315K- FAS1$^{opt;RK}$-FAS2^{opt}	*CEN6/ARS4, AmpR, kanMX4, pFAS1-FAS1$^{opt;R1834K}$-tFAS1, pFAS2-FAS2^{opt}-tFAS2*	S5	This study
FWV125	pRS315K- fusFAS$^{opt;RK}$	*CEN6/ARS4, AmpR, kanMX4, pFAS1-FAS1$^{opt;R1834K}$-FAS2-tFAS2*	S5	This study
FWV128	pRS313K-fusFAS^{RK}	*CEN6/ARS4, AmpR, kanMX4, pFAS1-FAS1 ^{R1834K} -FAS2-tFAS2*	Fig. 4a,b; S5	This study
FWV129	pRS315K-FAS1-FAS2	*CEN6/ARS4, AmpR, kanMX4, pFAS1-FAS1-tFAS1, pFAS2-FAS2-tFAS2*	Fig. 4b; S3	This study
FWV132	pRS313H-fusFAS	*CEN6/ARS4, AmpR, HphNT1, pFAS1-FAS1-FAS2-tFAS2*	Fig. 4b; S3	This study
FWV133	pRS313H-fusFAS^{RK}	*CEN6/ARS4, AmpR, HphNT1, pFAS1-FAS1^{R1834K}-FAS2-tFAS2*	Fig. 4b	This study
FWV155	pRS315H-FAS1^{RK} -FAS2	*CEN6/ARS4, AmpR, hphNT1, pFAS1-FAS1 ^{R1834K} -tFAS1, pFAS2-FAS2-tFAS2*	Fig. 4b	This study
SHV29	pRCC-N-kanMX4	*2μ, AmpR, natNT2, pROX3-opt.CAS9^{Sp}-tCYC1, pSNR52-gRNA[kanMX4]-tSUB4*	-	This study
SHV42	pRCC-N-faa2	*2μ, AmpR, natNT2, pROX3-opt.CAS9^{Sp}-tCYC1, pSNR52-gRNA[faa2]-tSUB4*	-	Henritzi et al., 2018
SHV69	pRS313-fusFAS	*CEN6/ARS4, AmpR, HIS3, pFAS1-FAS1-FAS2-tFAS2*	Fig. 2	This study
SHV70	pRS313-fusFAS^{RK}	*CEN6/ARS4, AmpR, HIS3, pFAS1-FAS1^{R1834K}-FAS2-tFAS2*	Fig. 2; Fig. 3; S4	This study
SHV72	pRS313-pHXT7-fusFAS^{RK}	*CEN6/ARS4, AmpR, HIS3, pHXT7^{-1-392}-FAS1^{R1834K}-FAS2-tFAS2*	Fig. 3	This study
SHV73	pRS313-pTEF1-fusFAS^{RK}	*CEN6/ARS4, AmpR, HIS3, pTEF1-FAS1^{R1834K}-FAS2-tFAS2*	Fig. 3	This study
SHV74	pRS313-pTDH3-fusFAS^{RK}	*CEN6/ARS4, AmpR, HIS3, pTDH3-FAS1^{R1834K}-FAS2-tFAS2*	Fig. 3	This study

Supplementary Table S2 | Oligonucleotides used in this study

Primer Name	Sequence 5′-3′	Application
FWP220	AGTTTATGTACAAATATCATAAAAAAAGAGAATCTTTCAGCGACATGGAGGCC	Amplification of *kanMX4* with overhangs for pRS315 backbone to replace *LEU2*
FWP221	TTCTCAACAAGTAATTGGTTGTTTGGCCGAGCGGTCTGACACTGGATGGCGGCG	
FWP263	CGGCATCAGAGCAGATTGTACTGAGAGTGCACCATCGTCCCAAAACCTTCTCAAG	Amplification of *hphNT1* with overhangs for pRS313 backbone to replace *HIS3*
FWP264	GCATCTGTGCGGTATTTCACACCGCATATGATCCGCAGCGACATGGAGGC	
FWP275	GGCATCAGAGCAGATTGTACTGAGAGTGCACCATAATTCCAGCGACATGGAGGCC	Amplification of *kanMX4* with overhangs for pRS313 backbone to replace *HIS3*
FWP276	TACGCATCTGTGCGGTATTTCACACCGCATATGATCCGTGACACTGGATGGCGGC	
FWP312	ATAAAGTTTATGTACAAATATCATAAAAAAAGAGAATCTTTCGTCCCAAAACCTTCTC	Amplification of *hphNT1* with overhangs for prS315 backbone to replace *LEU2*
FWP313	CTCTATTTCTCAACAAGTAATTGGTTGTTTGGCCGAGCGGTCTCAGCGACATGGAGG	
SHP100	ATGACCTTGATGATTAGCGAAGTAAGTCGACCTAGGTTCTATCCGAACTCCCCTTTTG	Amplification of pFAS2-FAS2-tFAS2 with overlaps to pRS315-FAS1-RK backbone
SHP101	CCCTCACTAAAGGGAACAAAAGCTGGGTACCGGGCATGCATAGAGCTGCTACGCGGAC	
SHP145	TGAATTGTAATACGACTCACTATAGGGCGAATTGGAGCTCCGCTCGTAGGAACAATTTCG	Amplification of *pHXT7*$^{-1--392}$ with overhangs for pRS313-fusFAS^{RK} backbone to replace *pFAS1*
SHP26	GTGAGATAGGGTTAATGGTCTTGTGGAGTAAGCGTCCATTTTTTTGATTAAAATTAAAAAAACTTTTTGTTT	
SHP150	GAATTGTAATACGACTCACTATAGGGCGAATTGGAGCTCCACAGTTTATTCCTGGCATCC	Amplification of *pTDH3* with overhangs for pRS313-fusFAS^{RK} backbone to replace *pFAS1*
SHP151	ATAGGGTTAATGGTCTTGTGGAGTAAGCGTCCATTTTGTTTGTTTATGTGTGTTTATTCG	
SHP152	TGAATTGTAATACGACTCACTATAGGGCGAATTGGAGCTCCCCTTGCCAACAGGGAGTTC	Amplification of *pTEF1* with overhangs for pRS313-fusFAS^{RK} backbone to replace *pFAS1*
SHP153	GGGTTAATGGTCTTGTGGAGTAAGCGTCCATTTTGTAATTAAAACTTAGATTAGATTGCTATGC	
SHP78	CTCATGACGAGCTCCGTATGCTAAATGAATGTACTTATGACGATTTGGAACACATTCAAA	Donor-DNA fragment for faa2 deletion
SHP62	CCAAGGGAAAAATATAAAAAAGTACATTGGGCCTTTTCATGGCGGCCGACTGGTTTCTTAAGTGATTATATAACCGTAAGAAATATTTAACTT	Donor-DNA fragment for *kanMX4* cassette deletion in *Δfas1* locus
SHP63	TTTTCACATGCTACCTCATTCGCCTCGTAACGTTACGACCGGGCCCCCTGCGGCTCCGGTAAGAGAGCACTACGTAGTCCCTCTTTTAATATGTAACGTGT	Donor-DNA fragment for *kanMX4* cassette deletion in *Δfas2* locus

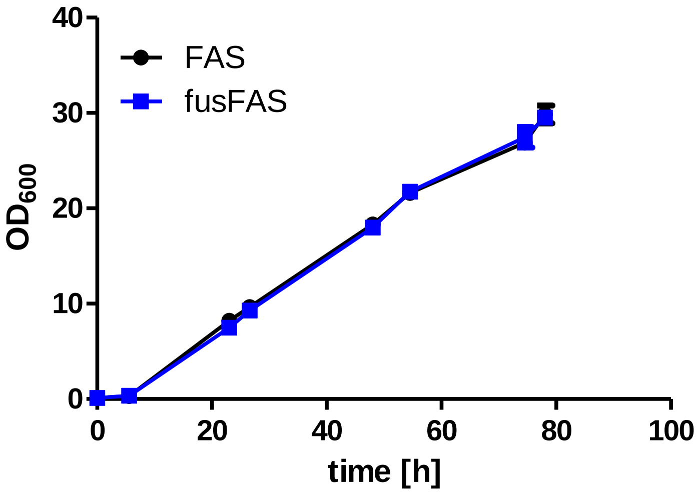

Supplementary Figure S3 | Complementation of FAS deficiency by FAS constructs. The wildtype split FAS (FAS) and fused FAS (fusFAS) were expressed in the strain SHY34 (*Δfas1 Δfas2 Δfaa2*) cultivated in buffered YPD medium. The growth was assessed by measuring OD_{600} over time. Mean values and standard deviations of biological duplicates are shown. Error bars may be smaller than the symbols.

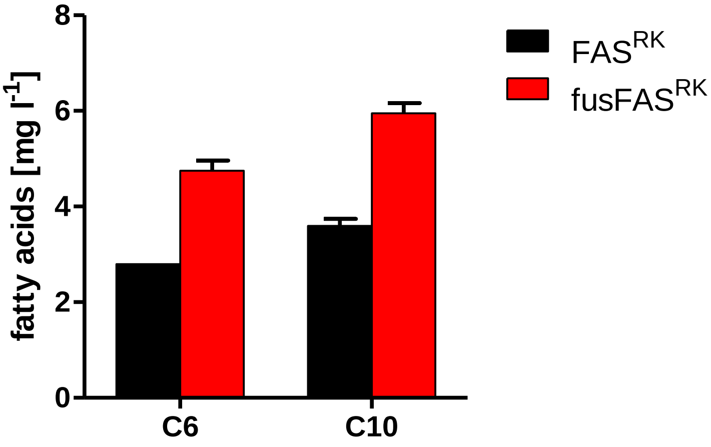

Supplementary Figure S4 | Production of other SMCFA by FAS constructs. The production of hexanoic acid (C6) and decanoic acid (C10) by two gene encoded FAS (FAS^{RK}) and fused FAS ($fusFAS^{RK}$) was quantified in the same samples (at 48 h) as shown in Fig. 2b in the main manuscript. Mean values and standard deviations of biological duplicates are shown.

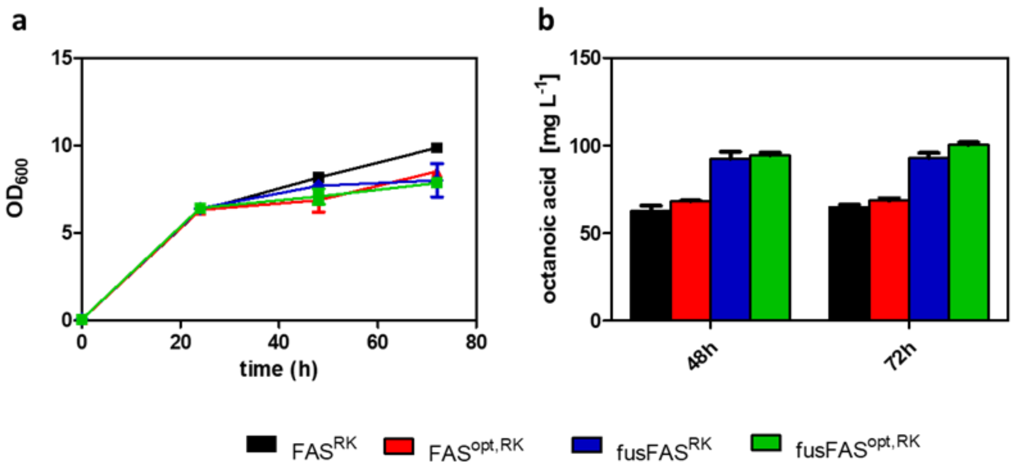

Supplementary Figure S5 | Codon-optimization of FAS-constructs. Mutated two gene encoded FAS (FAS^{RK}) and fused FAS ($fusFAS^{RK}$) were expressed in strain SHY34 (*Δfas1 Δfas2 Δfaa2*) as codon optimized (opt.) or non-optimized variants. Comparison of (a) growth) and octanoic acid production (b) in buffered YPD medium over a time of 72h. For plasmid maintenance hygromycin (100 mg l^{-1}) was used. Mean values and standard deviations of biological duplicates are shown.

Sequences of promoters and FAS constructs. Sequences of promoters used in Figure 3 are listed. The online tool JCat (http://www.jcat.de) was used to codon-optimize the nucleotide sequence and calculate the codon adaptation index (CAI) of FAS ORFs. The change in CAI resulting from optimization is indicated, where appropriate. To introduce the R1834K mutation, the respective native codon "AGA" in *FAS1* was replaced by "AAA".

```
>pFAS1
TGCGGTCTCGTCCTCTACGAATATGGCTATTTGCCTTCGTATATACCTTTCTATACCAAG
TAATGAATGTCTTGAGGGCCCGTATGGCCGCGCGAAGGCTTAGTTAAGATGTTTCAGCAA
ACGGCAGCATGTGAAAAAACCCGTAGAAGGTCCGCATCAGCCTTCCATGCCCGTGCACCC
ACGGCTCCTCGGAGGCCGGGTTATAGCAGCGTCTGCTCCGCATCACGATACACGAGGTGC
AGGCACGGTTCACTACTCCCCTGGCCTCCAACAAACGACGGCCAAAAACTTCACATGCCG
CCCAGCCAAGCATAATTACGCAACAGCGATCTTTCCGTCGCACAAGTTAAAAGAAATTGT
TGAAAAATACAAATAATCGCGAACAATACGTTGTTGCTATTTAACGCTTTTGGTCTGACA
GTAAGTGTGCCTTTCCCAATCACCGAAAAGTGTTGAACGATTCACTGCGACAATAATCAG
AGATTACAGTCGGCATTTTGGCATTTTTGGCATACTTTTTATCGATTGAACCATCTTCTC
CAAACACTTTTCCTTTTTCCTTCTATTCTGCAGGACCAACTAAAACTGGGTATATATATC
ATTATCTATATATATAAACGGCTTTCAACAAAGTTATAGGGGAAAACTAAAAATATAAGA
AAAAAAAAGGTATTGATTGATAAGGAAAAAGAACCAAGGGAAAAATATAAAAAAGTACAT
TGGGCCTTTTCATACTTGTTATCACTTACATTACAAAGAAGAACAAACAACTTTTTTAAA
CGAATTTTCTTTCTTCCTTTTTCAATTTATTAATTCTTTTTTTCCATACAATTCAAGGTC
AAATATATTCTTATATGCTCTTTGAATATTTCTGAAAAATATATAAAGAAAAGAAACTAC
AAGAACATCATCCGGAAAATCAGATTATAGACTAGGATTCCGCTCTTTTTAGTATATTTA
TTCGCCACACCTAACTGCTCTATTATTCGCTCATT
```

```
>pFAS2
TTCTATCCGAACTCCCCTTTTGTATATCAATATATCTTAATACTTTCGCCTATTCTTTAC
TATATTTCCTAAATTTTCTCTGGTCTGCAGGCCAAAAACAACAACTTACTACTGAATCAT
GGACGTGTATTTAGTTTAGCCAAGCAATATTTAAATATCACTCTTCCTAAAAATACATTG
GGCATTACCCGCAAACTAACCCATCGCTTAGCAAAATCCAACCATTTTTTTTTTATCTCC
CGCGTTTTCACATGCTACCTCATTCGCCTCGTAACGTTACGACCGAAATCTCACTAAGGC
ACGGTTTGTTGGGCAGTTTACAGATGTTGGATAACCAGTTGTTTCTAAACGGTTATGCCT
CATATATAACTTGTTAACTGAAGGTTACACAAGACCACATCACCACTGTCGTGCTTTTCT
AATAACCGCTATATTAGACGTTTAAAGGGCTACAGCAACACCAATTGAAATACCATCATT
```

```
>pTDH3
ACAGTTTATTCCTGGCATCCACTAAATATAATGGAGCCCGCTTTTTAAGCTGGCATCCAG
AAAAAAAAAGAATCCCAGCACCAAAATATTGTTTTCTTCACCAACCATCAGTTCATAGGT
CCATTCTCTTAGCGCAACTACAGAGAACAGGGGCACAAACAGGCAAAAAACGGGCACAAC
CTCAATGGAGTGATGCAACCTGCCTGGAGTAAATGATGACACAAGGCAATTGACCCACGC
ATGTATCTATCTCATTTTCTTACACCTTCTATTACCTTCTGCTCTCTCTGATTTGGAAAA
AGCTGAAAAAAAAGGTTGAAACCAGTTCCCTGAAATTATTCCCCTACTTGACTAATAAGT
ATATAAAGACGGTAGGTATTGATTGTAATTCTGTAAATCTATTTCTTAAACTTCTTAAAT
TCTACTTTTATAGTTAGTCTTTTTTTTAGTTTTAAAACACCAAGAACTTAGTTTCGAATA
AACACACATAAACAAACAAA
```

```
>pTEF1
CCTTGCCAACAGGGAGTTCTTCAGAGACATGGAGGCTCAAAACGAAATTATTGACAGCCT
AGACATCAATAGTCATACAACAGAAAGCGACCACCCAACTTTGGCTGATAATAGCGTATA
AACAATGCATACTTTGTACGTTCAAAATACAATGCAGTAGATATATTTATGCATATTACA
TATAATACATATCACATAGGAAGCAACAGGCGCGTTGGACTTTTAATTTTCGAGGACCGC
GAATCCTTACATCACACCCAATCCCCCACAAGTGATCCCCCACACACCATAGCTTCAAAA
TGTTTCTACTCCTTTTTTACTCTTCCAGATTTTCTCGGACTCCGCGCATCGCCGTACCAC
TTCAAAACACCCAAGCACAGCATACTAAATTTCCCCTCTTTCTTCCTCTAGGGTGTCGTT
AATTACCCGTACTAAAGGTTTGGAAAAGAAAAAAGACACCGCCTCGTTTCTTTTTCTTCG
TCGAAAAAGGCAATAAAAATTTTTATCACGTTTCTTTTTCTTGAAAATTTTTTTTTTTGA
TTTTTTTCTCTTTCGATGACCTCCCATTGATATTTAAGTTAATAAACGGTCATCAATTTC
TCAAGTTTCAGTTTCATTTTTCTTGTTCTATTACAACTTTTTTTACTTCTTGCTCATTAG
AAAGAAAGCATAGCAATCTAATCTAAGTTTTAATTACAAA
```

>pHXT7^{-1-392}
GCTCGTAGGAACAATTTCGGGCCCCTGCGTGTTCTTCTGAGGTTCATCTTTTACATTTGC
TTCTGCTGGATAATTTTCAGAGGCAACAAGGAAAAATTAGATGGCAAAAAGTCGTCTTTC
AAGGAAAAATCCCCACCATCTTTCGAGATCCCCTGTAACTTATTGGCAACTGAAAGAATG
AAAAGGAGGAAAATACAAAATATACTAGAACTGAAAAAAAAAAAGTATAAATAGAGACGA
TATATGCCAATACTTCACAATGTTCGAATCTATTCTTCATTTGCAGCTATTGTAAAATAA
TAAAACATCAAGAACAAACAAGCTCAACTTGTCTTTTCTAAGAACAAAGAATAAACACAA
AAACAAAAAGTTTTTTTAATTTTAATCAAAAA

>codon optimized FAS1, CAI change: 0.27 > 0.98
ATGGACGCTTACTCTACTAGACCATTGACTTTGTCTCACGGTTCTTTGGAACACGTTTTG
TTGGTTCCAACTGCTTCTTTCTTCATCGCTTCTCAATTGCAAGAACAATTCAACAAGATC
TTGCCAGAACCAACTGAAGGTTTCGCTGCTGACGACGAACCAACTACTCCAGCTGAATTG
GTTGGTAAGTTCTTGGGTTACGTTTCTTCTTTGGTTGAACCATCTAAGGTTGGTCAATTC
GACCAAGTTTTGAACTTGTGTTTGACTGAATTCGAAAACTGTTACTTGGAAGGTAACGAC
ATCCACGCTTTGGCTGCTAAGTTGTTGCAAGAAAACGACACTACTTTGGTTAAGACTAAG
GAATTGATCAAGAACTACATCACTGCTAGAATCATGGCTAAGAGACCATTCGACAAGAAG
TCTAACTCTGCTTTGTTCAGAGCTGTTGGTGAAGGTAACGCTCAATTGGTTGCTATCTTC
GGTGGTCAAGGTAACACTGACGACTACTTCGAAGAATTGAGAGACTTGTACCAAACTTAC
CACGTTTTGGTTGGTGACTTGATCAAGTTCTCTGCTGAAACTTTGTCTGAATTGATCAGA
ACTACTTTGGACGCTGAAAAGGTTTTCACTCAAGGTTTGAACATCTTGGAATGGTTGGAA
AACCCATCTAACACTCCAGACAAGGACTACTTGTTGTCTATCCCAATCTCTTGTCCATTG
ATCGGTGTTATCCAATTGGCTCACTACGTTGTTACTGCTAAGTTGTTGGGTTTCACTCCA
GGTGAATTGAGATCTTACTTGAAGGGTGCTACTGGTCACTCTCAAGGTTTGGTTACTGCT
GTTGCTATCGCTGAAACTGACTCTTGGGAATCTTTCTTCGTTTCTGTTAGAAAGGCTATC
ACTGTTTTGTTCTTCATCGGTGTTAGATGTTACGAAGCTTACCCAAACACTTCTTTGCCA
CCATCTATCTTGGAAGACTCTTTGGAAAACAACGAAGGTGTTCCATCTCCAATGTTGTCT
ATCTCTAACTTGACTCAAGAACAAGTTCAAGACTACGTTAACAAGACTAACTCTCACTTG
CCAGCTGGTAAGCAAGTTGAAATCTCTTTGGTTAACGGTGCTAAGAACTTGGTTGTTTCT
GGTCCACCACAATCTTTGTACGGTTTGAACTTGACTTTGAGAAAGGCTAAGGCTCCATCT
GGTTTGGACCAATCTAGAATCCCATTCTCTGAAAGAAAGTTGAAGTTCTCTAACAGATTC
TTGCCAGTTGCTTCTCCATTCCACTCTCACTTGTTGGTTCCAGCTTCTGACTTGATCAAC
AAGGACTTGGTTAAGAACAACGTTTCTTTCAACGCTAAGGACATCCAAATCCCAGTTTAC
GACACTTTCGACGGTTCTGACTTGAGAGTTTTGTCTGGTTCTATCTCTGAAAGAATCGTT
GACTGTATCATCAGATTGCCAGTTAAGTGGGAAACTACTACTCAATTCAAGGCTACTCAC
ATCTTGGACTTCGGTCCAGGTGGTGCTTCTGGTTTGGGTGTTTTGACTCACAGAAACAAG
GACGGTACTGGTGTTAGAGTTATCGTTGCTGGTACTTTGGACATCAACCCAGACGACGAC
TACGGTTTCAAGCAAGAAATCTTCGACGTTACTTCTAACGGTTTGAAGAAGAACCCAAAC
TGGTTGGAAGAATACCACCCAAAGTTGATCAAGAACAAGTCTGGTAAGATCTTCGTTGAA
ACTAAGTTCTCTAAGTTGATCGGTAGACCACCATTGTTGGTTCCAGGTATGACTCCATGT
ACTGTTTCTCCAGACTTCGTTGCTGCTACTACTAACGCTGGTTACACTATCGAATTGGCT
GGTGGTGGTTACTTCTCTGCTGCTGGTATGACTGCTGCTATCGACTCTGTTGTTTCTCAA
ATCGAAAAGGGTTCTACTTTCGGTATCAACTTGATCTACGTTAACCCATTCATGTTGCAA
TGGGGTATCCCATTGATCAAGGAATTGAGATCTAAGGGTTACCCAATCCAATTCTTGACT
ATCGGTGCTGGTGTTCCATCTTTGGAAGTTGCTTCTGAATACATCGAAACTTTGGGTTTG
AAGTACTTGGGTTTGAAGCCAGGTTCTATCGACGCTATCTCTCAAGTTATCAACATCGCT
AAGGCTCACCCAAACTTCCCAATCGCTTTGCAATGGACTGGTGGTAGAGGTGGTGGTCAC
CACTCTTTCGAAGACGCTCACACTCCAATGTTGCAAATGTACTCTAAGATCAGAAGACAC
CCAAACATCATGTTGATCTTCGGTTCTGGTTTCGGTTCTGCTGACGACACTTACCCATAC
TTGACTGGTGAATGGTCTACTAAGTTCGACTACCCACCAATGCCATTCGACGGTTTCTTG
TTCGGTTCTAGAGTTATGATCGCTAAGGAAGTTAAGACTTCTCCAGACGCTAAGAAGTGT
ATCGCTGCTTGTACTGGTGTTCCAGACGACAAGTGGGAACAAACTTACAAGAAGCCAACT
GGTGGTATCGTTACTGTTAGATCTGAAATGGGTGAACCAATCCACAAGATCGCTACTAGA
GGTGTTATGTTGTGGAAGGAATTCGACGAAACTATCTTCAACTTGCCAAAGAACAAGTTG
GTTCCAACTTTGGAAGCTAAGAGAGACTACATCATCTCTAGATTGAACGCTGACTTCCAA
AAGCCATGGTTCGCTACTGTTAACGGTCAAGCTAGAGACTTGGCTACTATGACTTACGAA
GAAGTTGCTAAGAGATTGGTTGAATTGATGTTCATCAGATCTACTAACTCTTGGTTCGAC
GTTACTTGGAGAACTTTCACTGGTGACTTCTTGAGAAGAGTTGAAGAAAGATTCACTAAG
TCTAAGACTTTGTCTTTGATCCAATCTTACTCTTTGTTGGACAAGCCAGACGAAGCTATC
GAAAAGGTTTTCAACGCTTACCCAGCTGCTAGAGAACAATTCTTGAACGCTCAAGACATC
GACCACTTCTTGTCTATGTGTCAAAACCCAATGCAAAAGCCAGTTCCATTCGTTCCAGTT
TTGGACAGAAGATTCGAAATCTTCTTCAAGAAGGACTCTTTGTGGCAATCTGAACACTTG

GAAGCTGTTGTTGACCAAGACGTTCAAAGAACTTGTATCTTGCACGGTCCAGTTGCTGCT
CAATTCACTAAGGTTATCGACGAACCAATCAAGTCTATCATGGACGGTATCCACGACGGT
CACATCAAGAAGTTGTTGCACCAATACTACGGTGACGACGAATCTAAGATCCCAGCTGTT
GAATACTTCGGTGGTGAATCTCCAGTTGACGTTCAATCTCAAGTTGACTCTTCTTCTGTT
TCTGAAGACTCTGCTGTTTTCAAGGCTACTTCTTCTACTGACGAAGAATCTTGGTTCAAG
GCTTTGGCTGGTTCTGAAATCAACTGGAGACACGCTTCTTTCTTGTGTTCTTTCATCACT
CAAGACAAGATGTTCGTTTCTAACCCAATCAGAAAGGTTTTCAAGCCATCTCAAGGTATG
GTTGTTGAAATCTCTAACGGTAACACTTCTTCTAAGACTGTTGTTACTTTGTCTGAACCA
GTTCAAGGTGAATTGAAGCCAACTGTTATCTTGAAGTTGTTGAAGGAAAACATCATCCAA
ATGGAAATGATCGAAAACAGAACTATGGACGGTAAGCCAGTTTCTTTGCCATTGTTGTAC
AACTTCAACCCAGACAACGGTTTCGCTCCAATCTCTGAAGTTATGGAAGACAGAAACCAA
AGAATCAAGGAAATGTACTGGAAGTTGTGGATCGACGAACCATTCAACTTGGACTTCGAC
CCAAGAGACGTTATCAAGGGTAAGGACTTCGAAATCACTGCTAAGGAAGTTTACGACTTC
ACTCACGCTGTTGGTAACAACTGTGAAGACTTCGTTTCTAGACCAGACAGAACTATGTTG
GCTCCAATGGACTTCGCTATCGTTGTTGGTTGGAGAGCTATCATCAAGGCTATCTTCCCA
AACACTGTTGACGGTGACTTGTTGAAGTTGGTTCACTTGTCTAACGGTTACAAGATGATC
CCAGGTGCTAAGCCATTGCAAGTTGGTGACGTTGTTTCTACTACTGCTGTTATCGAATCT
GTTGTTAACCAACCAACTGGTAAGATCGTTGACGTTGTTGGTACTTTGTCTAGAAACGGT
AAGCCAGTTATGGAAGTTACTTCTTCTTTCTTCTACAGAGGTAACTACACTGACTTCGAA
AACACTTTCCAAAAGACTGTTGAACCAGTTTACCAAATGCACATCAAGACTTCTAAGGAC
ATCGCTGTTTTGAGATCTAAGGAATGGTTCCAATTGGACGACGAAGACTTCGACTTGTTG
AACAAGACTTTGACTTTCGAAACTGAAACTGAAGTTACTTTCAAGAACGCTAACATCTTC
TCTTCTGTTAAGTGTTTCGGTCCAATCAAGGTTGAATTGCCAACTAAGGAAACTGTTGAA
ATCGGTATCGTTGACTACGAAGCTGGTGCTTCTCACGGTAACCCAGTTGTTGACTTCTTG
AAGAGAAACGGTTCTACTTTGGAACAAAAGGTTAACTTGGAAAACCCAATCCCAATCGCT
GTTTTGGACTCTTACACTCCATCTACTAACGAACCATACGCTAGAGTTTCTGGTGACTTG
AACCCAATCCACGTTTCTAGACACTTCGCTTCTTACGCTAACTTGCCAGGTACTATCACT
CACGGTATGTTCTCTTCTGCTTCTGTTAGAGCTTTGATCGAAAACTGGGCTGCTGACTCT
GTTTCTTCTAGAGTTAGAGGTTACACTTGTCAATTCGTTGACATGGTTTTGCCAAACACT
GCTTTGAAGACTTCTATCCAACACGTTGGTATGATCAACGGTAGAAAGTTGATCAAGTTC
GAAACTAGAAACGAAGACGACGTTGTTGTTTTGACTGGTGAAGCTGAAATCGAACAACCA
GTTACTACTTTCGTTTTCACTGGTCAAGGTTCTCAAGAACAAGGTATGGGTATGGACTTG
TACAAGACTTCTAAGGCTGCTCAAGACGTTTGGAACAGAGCTGACAACCACTTCAAGGAC
ACTTACGGTTTCTCTATCTTGGACATCGTTATCAACAACCCAGTTAACTTGACTATCCAC
TTCGGTGGTGAAAAGGGTAAGAGAATCAGAGAAAACTACTCTGCTATGATCTTCGAAACT
ATCGTTGACGGTAAGTTGAAGACTGAAAAGATCTTCAAGGAAATCAACGAACACTCTACT
TCTTACACTTTCAGATCTGAAAAGGGTTTGTTGTCTGCTACTCAATTCACTCAACCAGCT
TTGACTTTGATGGAAAAGGCTGCTTTCGAAGACTTGAAGTCTAAGGGTTTGATCCCAGCT
GACGCTACTTTCGCTGGTCACTCTTTGGGTGAATACGCTGCTTTGGCTTCTTTGGCTGAC
GTTATGTCTATCGAATCTTTGGTTGAAGTTGTTTTCTACAGAGGTATGACTATGCAAGTT
GCTGTTCCAAGAGACGAATTGGGTAGATCTAACTACGGTATGATCGCTATCAACCCAGGT
AGAGTTGCTGCTTCTTTCTCTCAAGAAGCTTTGCAATACGTTGTTGAAAGAGTTGGTAAG
AGAACTGGTTGGTTGGTTGAAATCGTTAACTACAACGTTGAAAACCAACAATACGTTGCT
GCTGGTGACTTGAGAGCTTTGGACACTGTTACTAACGTTTTGAACTTCATCAAGTTGCAA
AAGATCGACATCATCGAATTGCAAAAGTCTTTGTCTTTGGAAGAAGTTGAAGGTCACTTG
TTCGAAATCATCGACGAAGCTTCTAAGAAGTCTGCTGTTAAGCCAAGACCATTGAAGTTG
GAAAGAGGTTTCGCTTGTATCCCATTGGTTGGTATCTCTGTTCCATTCCACTCTACTTAC
TTGATGAACGGTGTTAAGCCATTCAAGTCTTTCTTGAAGAAGAACATCATCAAGGAAAAC
GTTAAGGTTGCTAGATTGGCTGGTAAGTACATCCCAAACTTGACTGCTAAGCCATTCCAA
GTTACTAAGGAATACTTCCAAGACGTTTACGACTTGACTGGTTCTGAACCAATCAAGGAA
ATCATCGACAACTGGGAAAAGTACGAACAATCTTAA

>codon optimized FAS2, CAI change: 0.32 > 0.98
ATGAAGCCAGAAGTTGAACAAGAATTGGCTCACATCTTGTTGACTGAATTGTTGGCTTAC
CAATTCGCTTCTCCAGTTAGATGGATCGAAACTCAAGACGTTTTCTTGAAGGACTTCAAC
ACTGAAAGAGTTGTTGAAATCGGTCCATCTCCAACTTTGGCTGGTATGGCTCAAAGAACT
TTGAAGAACAAGTACGAATCTTACGACGCTGCTTTGTCTTTGCACAGAGAAATCTTGTGT
TACTCTAAGGACGCTAAGGAAATCTACTACACTCCAGACCCATCTGAATTGGCTGCTAAG
GAAGAACCAGCTAAGGAAGAAGCTCCAGCTCCAACTCCAGCTGCTTCTGCTCCAGCTCCA
GCTGCTGCTGCTCCAGCTCCAGTTGCTGCTGCTGCTCCAGCTGCTGCTGCTGCTGAAATC
GCTGACGAACCAGTTAAGGCTTCTTTGTTGTTGCACGTTTTGGTTGCTCACAAGTTGAAG

AAGTCTTTGGACTCTATCCCAATGTCTAAGACTATCAAGGACTTGGTTGGTGGTAAGTCT
ACTGTTCAAAACGAAATCTTGGGTGACTTGGGTAAGGAATTCGGTACTACTCCAGAAAAG
CCAGAAGAAACTCCATTGGAAGAATTGGCTGAAACTTTCCAAGACACTTTCTCTGGTGCT
TTGGGTAAGCAATCTTCTTCTTTGTTGTCTAGATTGATCTCTTCTAAGATGCCAGGTGGT
TTCACTATCACTGTTGCTAGAAAGTACTTGCAAACTAGATGGGGTTTGCCATCTGGTAGA
CAAGACGGTGTTTTGTTGGTTGCTTTGTCTAACGAACCAGCTGCTAGATTGGGTTCTGAA
GCTGACGCTAAGGCTTTCTTGGACTCTATGGCTCAAAAGTACGCTTCTATCGTTGGTGTT
GACTTGTCTTCTGCTGCTTCTGCTTCTGGTGCTGCTGGTGCTGGTGCTGCTGCTGGTGCT
GCTATGATCGACGCTGGTGCTTTGGAAGAAATCACTAAGGACCACAAGGTTTTGGCTAGA
CAACAATTGCAAGTTTTGGCTAGATACTTGAAGATGGACTTGGACAACGGTGAAAGAAAG
TTCTTGAAGGAAAAGGACACTGTTGCTGAATTGCAAGCTCAATTGGACTACTTGAACGCT
GAATTGGGTGAATTCTTCGTTAACGGTGTTGCTACTTCTTTCTCTAGAAAGAAGGCTAGA
ACTTTCGACTCTTCTTGGAACTGGGCTAAGCAATCTTTGTTGTCTTTGTACTTCGAAATC
ATCCACGGTGTTTTGAAGAACGTTGACAGAGAAGTTGTTTCTGAAGCTATCAACATCATG
AACAGATCTAACGACGCTTTGATCAAGTTCATGGAATACCACATCTCTAACACTGACGAA
ACTAAGGGTGAAAACTACCAATTGGTTAAGACTTTGGGTGAACAATTGATCGAAAACTGT
AAGCAAGTTTTGGACGTTGACCCAGTTTACAAGGACGTTGCTAAGCCAACTGGTCCAAAG
ACTGCTATCGACAAGAACGGTAACATCACTTACTCTGAAGAACCAAGAGAAAAGGTTAGA
AAGTTGTCTCAATACGTTCAAGAAATGGCTTTGGGTGGTCCAATCACTAAGGAATCTCAA
CCAACTATCGAAGAAGACTTGACTAGAGTTTACAAGGCTATCTCTGCTCAAGCTGACAAG
CAAGACATCTCTTCTTCTACTAGAGTTGAATTCGAAAAGTTGTACTCTGACTTGATGAAG
TTCTTGGAATCTTCTAAGGAAATCGACCCATCTCAAACTACTCAATTGGCTGGTATGGAC
GTTGAAGACGCTTTGGACAAGGACTCTACTAAGGAAGTTGCTTCTTTGCCAAACAAGTCT
ACTATCTCTAAGACTGTTTCTTCTACTATCCCAAGAGAAACTATCCCATTCTTGCACTTG
AGAAAGAAGACTCCAGCTGGTGACTGGAAGTACGACAGACAATTGTCTTCTTTGTTCTTG
GACGGTTTGGAAAAGGCTGCTTTCAACGGTGTTACTTTCAAGGACAAGTACGTTTTGATC
ACTGGTGCTGGTAAGGGTTCTATCGGTGCTGAAGTTTTGCAAGGTTTGTTGCAAGGTGGT
GCTAAGGTTGTTGTTACTACTTCTAGATTCTCTAAGCAAGTTACTGACTACTACCAATCT
ATCTACGCTAAGTACGGTGCTAAGGGTTCTACTTTGATCGTTGTTCCATTCAACCAAGGT
TCTAAGCAAGACGTTGAAGCTTTGATCGAATTCATCTACGACACTGAAAAGAACGGTGGT
TTGGGTTGGGACTTGGACGCTATCATCCCATTCGCTGCTATCCCAGAACAAGGTATCGAA
TTGGAACACATCGACTCTAAGTCTGAATTCGCTCACAGAATCATGTTGACTAACATCTTG
AGAATGATGGGTTGTGTTAAGAAGCAAAAGTCTGCTAGAGGTATCGAAACTAGACCAGCT
CAAGTTATCTTGCCAATGTCTCCAAACCACGGTACTTTCGGTGGTGACGGTATGTACTCT
GAATCTAAGTTGTCTTTGGAAACTTTGTTCAACAGATGGCACTCTGAATCTTGGGCTAAC
CAATTGACTGTTTGTGGTGCTATCATCGGTTGGACTAGAGGTACTGGTTTGATGTCTGCT
AACAACATCATCGCTGAAGGTATCGAAAAGATGGGTGTTAGAACTTTCTCTCAAAAGGAA
ATGGCTTTCAACTTGTTGGGTTTGTTGACTCCAGAAGTTGTTGAATTGTGTCAAAAGTCT
CCAGTTATGGCTGACTTGAACGGTGGTTTGCAATTCGTTCCAGAATTGAAGGAATTCACT
GCTAAGTTGAGAAAGGAATTGGTTGAAACTTCTGAAGTTAGAAAGGCTGTTTCTATCGAA
ACTGCTTTGGAACACAAGGTTGTTAACGGTAACTCTGCTGACGCTGCTTACGCTCAAGTT
GAAATCCAACCAAGAGCTAACATCCAATTGGACTTCCCAGAATTGAAGCCATACAAGCAA
GTTAAGCAAATCGCTCCAGCTGAATTGGAAGGTTTGTTGGACTTGGAAAGAGTTATCGTT
GTTACTGGTTTCGCTGAAGTTGGTCCATGGGGTTCTGCTAGAACTAGATGGGAAATGGAA
GCTTTCGGTGAATTCTCTTTGGAAGGTTGTGTTGAAATGGCTTGGATCATGGGTTTCATC
TCTTACCACAACGGTAACTTGAAGGGTAGACCATACACTGGTTGGGTTGACTCTAAGACT
AAGGAACCAGTTGACGACAAGGACGTTAAGGCTAAGTACGAAACTTCTATCTTGGAACAC
TCTGGTATCAGATTGATCGAACCAGAATTGTTCAACGGTTACAACCCAGAAAAGAAGGAA
ATGATCCAAGAAGTTATCGTTGAAGAAGACTTGGAACCATTCGAAGCTTCTAAGGAAACT
GCTGAACAATTCAAGCACCAACACGGTGACAAGGTTGACATCTTCGAAATCCCAGAAACT
GGTGAATACTCTGTTAAGTTGTTGAAGGGTGCTACTTTGTACATCCCAAAGGCTTTGAGA
TTCGACAGATTGGTTGCTGGTCAAATCCCAACTGGTTGGAACGCTAAGACTTACGGTATC
TCTGACGACATCATCTCTCAAGTTGACCCAATCACTTTGTTCGTTTTGGTTTCTGTTGTT
GAAGCTTTCATCGCTTCTGGTATCACTGACCCATACGAAATGTACAAGTACGTTCACGTT
TCTGAAGTTGGTAACTGTTCTGGTTCTGGTATGGGTGGTGTTTCTGCTTTGAGAGGTATG
TTCAAGGACAGATTCAAGGACGAACCAGTTCAAAACGACATCTTGCAAGAATCTTTCATC
AACACTATGTCTGCTTGGGTTAACATGTTGTTGATCTCTTCTTCTGGTCCAATCAAGACT
CCAGTTGGTGCTTGTGCTACTTCTGTTGAATCTGTTGACATCGGTGTTGAAACTATCTTG
TCTGGTAAGGCTAGAATCTGTATCGTTGGTGGTTACGACGACTTCCAAGAAGAAGGTTCT
TTCGAATTCGGTAACATGAAGGCTACTTCTAACACTTTGGAAGAATTCGAACACGGTAGA
ACTCCAGCTGAAATGTCTAGACCAGCTACTACTACTAGAAACGGTTTCATGGAAGCTCAA
GGTGCTGGTATCCAAATCATCATGCAAGCTGACTTGGCTTTGAAGATGGGTGTTCCAATC

TACGGTATCGTTGCTATGGCTGCTACTGCTACTGACAAGATCGGTAGATCTGTTCCAGCT
CCAGGTAAGGGTATCTTGACTACTGCTAGAGAACACCACTCTTCTGTTAAGTACGCTTCT
CCAAACTTGAACATGAAGTACAGAAAGAGACAATTGGTTACTAGAGAAGCTCAAATCAAG
GACTGGGTTGAAAACGAATTGGAAGCTTTGAAGTTGGAAGCTGAAGAAATCCCATCTGAA
GACCAAAACGAATTCTTGTTGGAAAGAACTAGAGAAATCCACAACGAAGCTGAATCTCAA
TTGAGAGCTGCTCAACAACAATGGGGTAACGACTTCTACAAGAGAGACCCAAGAATCGCT
CCATTGAGAGGTGCTTTGGCTACTTACGGTTTGACTATCGACGACTTGGGTGTTGCTTCT
TTCCACGGTACTTCTACTAAGGCTAACGACAAGAACGAATCTGCTACTATCAACGAAATG
ATGAAGCACTTGGGTAGATCTGAAGGTAACCCAGTTATCGGTGTTTTCCAAAAGTTCTTG
ACTGGTCACCCAAAGGGTGCTGCTGGTGCTTGGATGATGAACGGTGCTTTGCAAATCTTG
AACTCTGGTATCATCCCAGGTAACAGAAACGCTGACAACGTTGACAAGATCTTGGAACAA
TTCGAATACGTTTTGTACCCATCTAAGACTTTGAAGACTGACGGTGTTAGAGCTGTTTCT
ATCACTTCTTTCGGTTTCGGTCAAAAGGGTGGTCAAGCTATCGTTGTTCACCCAGACTAC
TTGTACGGTGCTATCACTGAAGACAGATACAACGAATACGTTGCTAAGGTTTCTGCTAGA
GAAAAGTCTGCTTACAAGTTCTTCCACAACGGTATGATCTACAACAAGTTGTTCGTTTCT
AAGGAACACGCTCCATACACTGACGAATTGGAAGAAGACGTTTACTTGGACCCATTGGCT
AGAGTTTCTAAGGACAAGAAGTCTGGTTCTTTGACTTTCAACTCTAAGAACATCCAATCT
AAGGACTCTTACATCAACGCTAACACTATCGAAACTGCTAAGATGATCGAAAACATGACT
AAGGAAAAGGTTTCTAACGGTGGTGTTGGTGTTGACGTTGAATTGATCACTTCTATCAAC
GTTGAAAACGACACTTTCATCGAAAGAAACTTCACTCCACAAGAAATCGAATACTGTTCT
GCTCAACCATCTGTTCAATCTTCTTTCGCTGGTACTTGGTCTGCTAAGGAAGCTGTTTTC
AAGTCTTTGGGTGTTAAGTCTTTGGGTGGTGGTGCTGCTTTGAAGGACATCGAAATCGTT
AGAGTTAACAAGAACGCTCCAGCTGTTGAATTGCACGGTAACGCTAAGAAGGCTGCTGAA
GAAGCTGGTGTTACTGACGTTAAGGTTTCTATCTCTCACGACGACTTGCAAGCTGTTGCT
GTTGCTGTTTCTACTAAGAAGTAA

>fusFAS based on native FAS1 and FAS2 ORFs
ATGGACGCTTACTCCACAAGACCATTAACCCTATCTCACGGTTCTTTAGAGCACGTGCTT
CTGGTACCAACCGCTTCATTTTTCATTGCTTCGCAATTACAAGAACAATTTAATAAAATT
TTGCCCGAACCCACTGAAGGGTTTGCTGCAGATGACGAGCCTACCACACCTGCTGAACTA
GTGGGGAAATTCCTTGGCTACGTATCTTCTCTAGTCGAACCTTCCAAGGTCGGTCAATTC
GATCAGGTCTTGAACCTTTGCTTAACAGAATTTGAAAACTGTTATTTAGAAGGCAATGAC
ATTCACGCCTTGGCTGCTAAACTATTACAGGAAAACGACACAACTTTAGTGAAGACTAAA
GAACTAATTAAAAATTATATTACCGCCAGAATAATGGCTAAGAGACCATTTGACAAAAAA
TCCAACTCTGCTCTTTTTAGGGCCGTCGGCGAGGGTAACGCACAATTGGTAGCCATTTTC
GGTGGTCAAGGTAACACCGACGACTACTTTGACGAATTGCGTGATCTATATCAAACTTAT
CATGTCTTAGTGGGAGATTTAATCAAGTTCTCCGCTGAAACTTTAAGTGAACTGATTAGA
ACTACTTTAGATGCTGAAAAAGTCTTTACTCAAGGTTTAAACATATTGGAATGGTTGGAG
AACCCTTCAAATACCCCAGACAAGGACTATTTACTTTCCATTCCAATTTCATGTCCCTTA
ATTGGTGTCATTCAATTGGCTCACTACGTAGTTACTGCCAAGCTTTTGGGTTTCACTCCA
GGTGAGTTAAGATCTTACTTAAAAGGTGCTACAGGTCACTCTCAAGGTTTGGTTACTGCT
GTCGCCATAGCTGAGACGGATTCCTGGGAATCCTTCTTCGTCTCCGTAAGAAAGGCAATT
ACTGTATTATTCTTCATCGGTGTTCGTTGTTACGAAGCATACCCAAACACTTCCCTACCA
CCATCCATCTTGGAAGATTCCTTGGAAAACAATGAAGGTGTTCCATCTCCAATGTTGTCC
ATTTCCAATCTAACTCAAGAACAAGTTCAAGACTATGTAAATAAGACTAACTCTCATTTG
CCAGCTGGTAAACAAGTTGAAATTTCTCTAGTCAATGGTGCGAAGAATCTAGTCGTATCG
GGCCCACCACAATCATTATATGGTTTAAACTTGACTTTAAGAAAGGCCAAGGCCCCATCT
GGACTGGATCAATCAAGAATCCCATTCAGCGAAAGAAAATTGAAGTTCTCCAATAGGTTC
TTACCTGTTGCATCACCATTCCATTCCCATCTATTGGTTCCAGCTTCAGATTTGATTAAC
AAAGACTTAGTCAAAAACAATGTCAGCTTTAACGCTAAAGATATTCAAATCCCCGTTTAC
GACACTTTTGATGGTTCAGATCTAAGAGTCCTTTCAGGTTCCATTTCCGAGAGAATCGTC
GACTGCATCATTAGATTACCTGTCAAATGGGAAACTACTACACAATTCAAAGCCACCCAC
ATATTAGACTTTGGTCCAGGTGGAGCTTCCGGTTTAGGTGTTTTAACCCATCGTAATAAA
GATGGTACTGGTGTTCGTGTTATCGTTGCCGGTACTCTCGACATTAACCCAGATGATGAT
TACGGATTCAAGCAAGAAATCTTTGATGTTACTAGTAATGGTTTGAAGAAAAATCCAAAC
TGGTTGGAAGAATACCATCCAAAATTAATTAAGAACAAATCAGGCAAAATTTTTGTCGAA
ACAAAATTTTCTAAATTAATCGGTAGACCACCTTTATTGGTTCCTGGTATGACACCATGT
ACTGTTTCTCCAGATTTCGTAGCTGCTACCACAAATGCTGGTTATACCATTGAGTTGGCC
GGTGGTGGTTACTTTTCCGCAGCAGGTATGACCGCCGCTATTGATTCTGTGGTTTCTCAG
ATAGAAAAGGGTAGTACCTTCGGTATCAACTTGATCTACGTCAATCCATTTATGTTACAA
TGGGGTATTCCATTAATCAAGGAACTAAGAAGCAAAGGTTATCCAATTCAATTCTTGACC

ATTGGTGCTGGTGTCCCATCATTGGAAGTTGCTAGTGAATACATAGAGACATTAGGTTTG
AAGTACTTGGGTTTGAAACCAGGTTCCATTGATGCTATTTCGCAAGTTATAAACATTGCT
AAAGCACATCCAAACTTCCCAATAGCTTTACAATGGACCGGTGGTAGAGGTGGTGGTCAT
CATTCTTTCGAAGATGCCCACACTCCAATGTTACAAATGTACTCCAAGATTAGAAGACAT
CCAAACATTATGTTGATATTCGGTTCTGGTTTCGGTTCTGCTGATGACACTTACCCATAC
TTAACCGGTGAATGGTCCACAAAATTCGATTATCCACCAATGCCATTCGATGGTTTCCTA
TTTGGTTCGAGGGTCATGATTGCTAAGGAAGTTAAAACTTCTCCTGATGCTAAGAAGTGT
ATTGCTGCTTGTACTGGTGTTCCTGATGATAAATGGGAACAAACCTACAAGAAGCCAACT
GGTGGTATTGTCACTGTTCGCTCTGAAATGGGTGAACCAATTCACAAAATTGCCACTCGT
GGTGTTATGCTATGGAAGGAATTCGACGAAACCATCTTCAACTTACCAAAGAATAAGTTG
GTACCAACTTTGGAAGCAAAGAGAGATTACATTATCTCAAGATTGAACGCCGATTTCCAA
AAACCATGGTTTGCTACCGTCAACGGTCAAGCCCGTGACCTAGCCACAATGACATACGAA
GAAGTTGCAAAGAGATTGGTGGAATTAATGTTCATCAGATCTACCAACTCTTGGTTTGAT
GTCACATGGAGAACCTTTACTGGTGATTTCCTACGTCGTGTCGAAGAACGTTTCACTAAA
AGTAAGACATTGTCTTTAATCCAATCCTATTCTCTACTAGACAAGCCTGATGAAGCTATT
GAAAAAGTATTTAATGCTTATCCTGCCGCTAGGGAACAGTTCTTGAATGCGCAAGATATT
GATCACTTTTTGAGCATGTGTCAAAATCCAATGCAAAAACCAGTGCCTTTTGTTCCAGTT
TTGGATCGTAGATTCGAGATTTTTTTCAAAAAAGATTCGTTATGGCAATCTGAGCACTTG
GAAGCCGTCGTCGACCAAGACGTTCAAAGAACATGTATCCTACATGGACCTGTTGCAGCA
CAATTCACTAAAGTCATCGATGAACCAATTAAGAGCATTATGGATGGTATTCACGATGGT
CACATCAAAAAGTTACTACATCAATATTACGGTGACGATGAGTCAAAGATTCCAGCAGTT
GAGTACTTTGGTGGTGAAAGCCCTGTAGACGTACAAAGTCAAGTTGATTCTTCCTCTGTA
TCTGAAGACTCAGCTGTTTTTAAGGCAACATCCTCTACTGATGAAGAAAGCTGGTTTAAG
GCTTTGGCGGGATCCGAAATTAACTGGAGACATGCAAGTTTCTTATGTTCCTTTATCACT
CAAGATAAAATGTTTGTTTCTAACCCAATTAGAAAAGTTTTCAAGCCAAGCCAAGGAATG
GTTGTTGAGATTTCCAACGGCAATACTTCTTCAAAGACTGTTGTCACTCTTTCAGAACCT
GTTCAAGGTGAATTGAAACCAACTGTTATTTTGAAGTTGTTGAAGGAGAACATAATCCAA
ATGGAAATGATTGAGAACAGAACTATGGATGGTAAGCCCGTCAGCTTGCCATTGTTGTAC
AACTTCAACCCAGATAATGGTTTTGCTCCAATCTCTGAAGTTATGGAGGACAGAAACCAA
AGAATTAAGGAAATGTACTGGAAATTATGGATTGATGAGCCTTTCAATTTGGACTTTGAC
CCAAGAGATGTCATTAAGGGCAAAGATTTCGAGATCACCGCTAAAGAAGTTTATGACTTT
ACACACGCTGTTGGAAACAATTGTGAAGACTTCGTTTCTAGACCTGATAGAACGATGTTG
GCCCCAATGGACTTTGCTATTGTTGTCGGATGGAGAGCCATCATCAAGGCCATTTTCCCT
AATACGGTCGATGGTGACTTATTGAAGTTGGTTCATTTGTCTAACGGCTACAAGATGATT
CCTGGCGCTAAGCCACTGCAAGTTGGTGATGTTGTTTCAACTACTGCTGTTATTGAATCT
GTCGTCAACCAACCTACAGGAAAGATTGTCGATGTGGTAGGTACATTATCGAGAAATGGC
AAGCCTGTCATGGAAGTCACCTCCTCATTCTTCTACAGAGGCAACTATACTGACTTTGAA
AACACTTTCCAAAAGACTGTTGAACCTGTTTATCAAATGCACATCAAAACTTCTAAAGAT
ATAGCTGTCTTGCGCTCTAAGGAGTGGTTCCAATTGGACGATGAAGACTTCGATCTGTTA
AACAAAACTTTGACTTTCGAAACTGAAACTGAAGTTACTTTCAAGAATGCTAACATCTTC
TCTTCAGTGAAATGTTTTGGCCCAATTAAAGTTGAATTGCCAACCAAAGAAACCGTGGAG
ATCGGTATTGTCGATTACGAAGCCGGTGCCTCTCACGGTAACCCTGTTGTTGATTTCTTG
AAGAGAAACGGTTCCACATTGGAACAAAAGGTCAATCTAGAAAATCCTATTCCAATTGCA
GTACTTGATTCGTACACTCCAAGTACCAACGAACCATACGCTAGAGTTTCTGGTGATTTG
AATCCAATTCACGTTTCACGTCATTTTGCCTCTTACGCAAACTTGCCAGGTACTATCACG
CACGGTATGTTTTCTTCTGCTTCCGTCCGTGCTTTGATTGAAAACTGGGCTGCTGACAGT
GTTTCATCCAGGGTACGTGGCTACACTTGTCAATTTGTTGACATGGTTTTGCCTAACACT
GCTTTGAAAACATCGATTCAACATGTTGGTATGATCAATGGTAGAAAATTGATAAAGTTT
GAAACTAGAAATGAAGATGACGTTGTAGTTTTGACTGGTGAAGCCGAAATTGAACAACCT
GTTACTACCTTCGTTTTCACTGGTCAAGGTTCACAAGAACAAGGTATGGGTATGGACTTA
TACAAAACTTCTAAAGCTGCTCAAGATGTTTGGAATAGAGCTGACAACCATTTCAAGGAC
ACTTATGGTTTCTCTATCTTAGACATTGTCATTAACAACCCAGTTAACTTAACAATTCAC
TTCGGTGGTGAAAAGGGTAAGAGGATCAGAGAAAACTATTCTGCTATGATCTTTGAGACT
ATCGTGGATGGAAAATTGAAGACTGAAAAAATTTTCAAGGAAATTAATGAGCACAGTACT
TCTTACACATTTAGATCTGAAAAAGGTTTATTGTCTGCTACTCAATTTACACAACCAGCT
TTAACTTTGATGGAAAAAGCTGCTTTCGAAGACTTGAAATCTAAAGGTTTGATCCCAGCC
GATGCTACTTTTGCTGGTCACTCTTTAGGTGAGTATGCTGCTTTGGCCTCTTTGGCTGAT
GTTATGTCTATCGAATCTTTAGTTGAAGTTGTGTTCTACAGAGGTATGACTATGCAAGTT
GCTGTTCCAAGAGATGAGTTGGGCAGATCCAACTATGGTATGATTGCCATTAACCCAGGT
AGAGTCGCTGCATCATTCTCTCAAGAAGCTTTGCAATATGTTGTTGAGAGAGTTGGTAAG
AGAACCGGCTGGTTGGTTGAAATCGTCAACTACAACGTTGAAAACCAACAATATGTTGCA
GCTGGTGATCTAAGAGCTTTAGACACCGTTACCAATGTTCTAAACTTCATCAAATTACAA

AAAATTGATATTATTGAACTACAAAAGTCCTTATCTTTGGAAGAAGTTGAAGGTCATTTG
TTTGAGATCATTGACGAAGCTTCCAAGAAATCTGCTGTCAAGCCTCGCCCACTTAAATTG
GAGAGAGGTTTTGCTTGTATCCCATTAGTTGGTATTTCTGTTCCTTTCCATTCCACCTAC
TTGATGAATGGTGTTAAACCATTCAAGAGTTTCTTGAAGAAGAATATCATAAAAGAAAAT
GTGAAGGTTGCTAGATTGGCCGGAAAGTACATTCCAAACTTGACTGCAAAACCATTCCAG
GTTACTAAGGAATATTTCCAGGACGTTTATGATTTGACTGGCTCCGAACCTATCAAGGAA
ATCATCGACAACTGGGAAAAGGATGAATGGGGCAGCCCGGAAGTTGAGCAAGAATTAGCT
CATATTTTGCTAACTGAATTGTTAGCTTATCAATTTGCCTCTCCTGTGAGATGGATTGAA
ACTCAAGATGTTTTTTTGAAGGATTTTAACACTGAAAGGGTTGTTGAAATCGGTCCTTCT
CCAACTTTGGCTGGGATGGCTCAAAGAACCTTGAAGAATAAATACGAATCTTACGATGCT
GCTCTGTCTTTACATAGAGAAATCTTATGCTATTCGAAGGATGCCAAAGAGATTTATTAT
ACCCCAGATCCATCCGAACTAGCTGCAAAGGAAGAGCCCGCTAAGGAAGAAGCTCCTGCT
CCAACTCCAGCTGCTAGTGCTCCTGCTCCTGCAGCAGCAGCCCCAGCTCCCGTCGCGGCA
GCAGCCCCAGCTGCAGCAGCTGCTGAGATTGCCGATGAACCTGTCAAGGCTTCCCTATTG
TTGCACGTTTTGGTTGCTCACAAGTTGAAGAAGTCGTTAGATTCCATTCCAATGTCCAAG
ACAATCAAAGACTTGGTCGGTGGTAAATCTACAGTCCAAAATGAAATTTTGGGTGATTTA
GGTAAAGAATTTGGTACTACTCCTGAAAAACCAGAAGAAACTCCATTAGAAGAATTGGCA
GAAACTTTCCAAGATACCTTCTCTGGAGCATTGGGTAAGCAATCTTCCTCGTTATTATCA
AGATTAATCTCATCTAAGATGCCTGGTGGGTTTACTATTACTGTCGCTAGAAAATACTTA
CAAACTCGCTGGGGACTACCATCTGGTAGACAAGATGGTGTCCTTTTGGTAGCTTTATCT
AACGAGCCTGCTGCTCGTCTAGGTTCTGAAGCTGATGCCAAGGCTTTCTTGGACTCCATG
GCTCAAAAATACGCTTCCATTGTTGGTGTTGACTTATCATCAGCTGCTAGCGCTAGTGGT
GCTGCCGGTGCAGGTGCTGCTGCCGGTGCAGCTATGATCGATGCTGGCGCTCTGGAAGAA
ATAACCAAAGACCACAAGGTTTTGGCGCGTCAACAACTGCAAGTATTGGCTCGTTATCTA
AAAATGGACTTGGATAACGGTGAAAGAAAGTTCTTGAAAGAAAAGGACACTGTTGCTGAA
CTTCAAGCTCAGTTGGATTACTTGAATGCCGAATTAGGTGAATTCTTTGTTAACGGTGTT
GCTACTTCTTTCTCTAGAAAAAAGGCCAGAACCTTCGATTCTTCCTGGAACTGGGCTAAA
CAATCTTTATTATCATTATACTTTGAGATAATTCATGGTGTCTTGAAAAACGTTGATAGA
GAGGTTGTTAGTGAAGCTATCAATATCATGAACAGATCTAACGATGCTTTGATTAAATTC
ATGGAATACCATATCTCTAACACTGATGAAACAAAAGGTGAAAACTATCAATTGGTTAAA
ACTCTTGGTGAGCAGTTGATTGAAAACTGTAAACAAGTTTTGGATGTTGATCCAGTTTAC
AAAGATGTTGCTAAGCCTACCGGTCCAAAAACTGCTATTGACAAGAACGGTAACATTACA
TACTCAGAAGAGCCAAGAGAAAAGGTTAGGAAATTATCTCAATACGTACAAGAAATGGCC
CTTGGTGGTCCAATCACCAAAGAATCTCAACCTACTATTGAAGAGGATTTGACTCGTGTT
TACAAGGCAATCAGTGCTCAAGCTGATAAACAAGATATTTCCAGCTCCACCAGGGTTGAA
TTTGAAAAACTATATAGTGATTTGATGAAGTTCTTGGAAAGCTCCAAAGAAATCGATCCT
TCTCAAACAACCCAATTGGCCGGTATGGATGTTGAGGATGCTTTGGACAAAGATTCCACC
AAAGAAGTTGCTTCTTTGCCAAACAAATCTACCATTTCTAAGACGGTATCTTCAACTATT
CCAAGAGAAACTATTCCGTTCTTACATTTGAGAAAGAAGACTCCTGCCGGAGATTGGAAA
TATGACCGCCAATTGTCTTCTCTTTTCTTAGATGGTTTAGAAAAGGCTGCCTTCAACGGT
GTCACCTTCAAGGACAAATACGTCTTGATCACTGGTGCTGGTAAGGGTTCTATTGGTGCT
GAAGTCTTGCAAGGTTTGTTACAAGGTGGTGCTAAGGTTGTTGTTACCACCTCTCGTTTC
TCTAAGCAAGTTACAGACTACTACCAATCCATTTACGCCAAATATGGTGCTAAGGGTTCT
ACTTTGATTGTTGTTCCATTCAACCAAGGTTCTAAGCAAGACGTTGAAGCTTTGATTGAA
TTTATCTACGACACTGAAAAGAATGGTGGTTTAGGTTGGGATCTAGATGCTATTATTCCA
TTCGCGGCCATTCCAGAACAAGGTATTGAATTAGAACATATTGATTCTAAGTCTGAATTT
GCTCATAGAATCATGTTGACCAATATCTTAAGAATGATGGGTTGTGTCAAGAAGCAAAAA
TCTGCAAGAGGTATTGAAACAAGACCAGCTCAAGTCATTCTACCAATGTCTCCAAACCAT
GGTACTTTCGGTGGTGATGGTATGTATTCAGAATCCAAGTTGTCTTTGGAAACTTTGTTC
AACAGATGGCACTCTGAATCCTGGGCCAATCAATTAACCGTTTGCGGTGCTATTATTGGT
TGGACTAGAGGTACTGGTTTAATGAGCGCTAATAACATCATTGCTGAAGGCATTGAAAAG
ATGGGTGTTCGTACTTTCTCTCAAAAGGAAATGGCTTTCAACTTATTGGGTCTATTGACT
CCAGAAGTCGTAGAATTGTGCCAAAAATCACCTGTTATGGCTGACTTGAATGGTGGTTTG
CAATTTGTTCCTGAATTGAAGGAATTCACTGCTAAATTGCGTAAAGAGTTGGTTGAAACT
TCTGAAGTTAGAAAGGCAGTTTCCATCGAAACTGCTTTGGAGCATAAGGTTGTCAATGGC
AATAGCGCTGATGCTGCATATGCTCAAGTCGAAATTCAACCAAGAGCTAACATTCAACTG
GACTTCCCAGAATTGAAACCATACAAACAGGTTAAACAAATTGCTCCCGCTGAGCTTGAA
GGTTTGTTGGATTTGGAAAGAGTTATTGTAGTTACCGGTTTTGCTGAAGTCGGCCCATGG
GGTTCGGCCAGAACAAGATGGGAAATGGAAGCTTTTGGTGAATTTTCGTTGGAAGGTTGC
GTTGAAATGGCCTGGATTATGGGCTTCATTTCATACCATAACGGTAATTTGAAGGGTCGT
CCATACACTGGTTGGGTTGATTCCAAAACAAAAGAACCAGTTGATGACAAGGACGTTAAG
GCCAAGTATGAAACATCAATCCTAGAACACAGTGGTATCAGATTGATCGAACCAGAGTTA

TTCAATGGTTACAACCCAGAAAAGAAGGAAATGATTCAAGAAGTCATTGTCGAAGAAGAC
TTGGAACCATTTGAGGCTTCGAAGGAAACTGCCGAACAATTTAAACACCAACATGGTGAC
AAAGTGGATATCTTCGAAATCCCAGAAACAGGAGAGTACTCTGTTAAGTTACTAAAGGGT
GCCACTTTATACATTCCAAAGGCTTTGAGATTTGACCGTTTGGTTGCAGGTCAAATTCCA
ACTGGTTGGAATGCTAAGACTTATGGTATCTCTGATGATATCATTTCTCAGGTTGACCCA
ATCACATTATTCGTTTTGGTCTCTGTTGTGGAAGCATTTATTGCATCTGGTATCACCGAC
CCATACGAAATGTACAAATACGTACATGTTTCTGAGGTTGGTAACTGTTCTGGTTCTGGT
ATGGGTGGTGTTTCTGCCTTACGTGGTATGTTTAAGGACCGTTTCAAGGATGAGCCTGTC
CAAAATGATATTTTACAAGAATCATTTATCAACACCATGTCCGCTTGGGTTAATATGTTG
TTGATTTCCTCATCTGGTCCAATCAAGACACCTGTTGGTGCCTGTGCCACATCCGTGGAA
TCTGTTGACATTGGTGTAGAAACCATCTTGTCTGGTAAGGCTAGAATCTGTATTGTCGGT
GGTTACGATGATTTCCAAGAAGAAGGCTCCTTTGAGTTCGGTAACATGAAGGCCACTTCC
AACACTTTGGAAGAATTTGAACATGGTCGTACCCCAGCGGAAATGTCCAGACCTGCCACC
ACTACCCGTAACGGTTTTATGGAAGCTCAAGGTGCTGGTATTCAAATCATCATGCAAGCT
GATTTAGCTTTGAAGATGGGTGTGCCAATTTACGGTATTGTTGCCATGGCTGCTACCGCC
ACCGATAAGATTGGTAGATCTGTGCCAGCTCCAGGTAAGGGTATTTTAACCACTGCTCGT
GAACACCACTCCAGTGTTAAGTATGCTTCACCAAACTTGAACATGAAGTACAGAAAGCGC
CAATTGGTTACTCGTGAAGCTCAGATTAAAGATTGGGTAGAAAACGAATTGGAAGCTTTG
AAGTTGGAGGCCGAAGAAATTCCAAGCGAAGACCAAAACGAGTTCTTACTTGAACGTACC
AGAGAAATCCACAACGAAGCTGAAAGTCAATTGAGAGCTGCACAACAACAATGGGGTAAC
GACTTCTACAAGAGGGACCCACGTATTGCTCCATTGAGAGGAGCACTGGCTACTTACGGT
TTAACTATTGATGACTTGGGTGTCGCTTCATTCCACGGTACATCCACAAAGGCTAATGAC
AAGAACGAATCTGCCACAATTAATGAAATGATGAAGCATTTGGGTAGATCTGAAGGTAAT
CCCGTCATTGGTGTTTTCCAAAAGTTCTTGACTGGTCATCCAAAGGGTGCTGCTGGTGCA
TGGATGATGAATGGTGCTTTGCAAATTCTAAACAGTGGTATTATTCCAGGTAACCGTAAC
GCTGATAACGTGGATAAGATCTTGGAGCAATTTGAATACGTCTTGTACCCATCCAAGACT
TTAAAGACCGACGGTGTCAGAGCCGTGTCCATCACTTCTTTCGGTTTTGGTCAAAAGGGT
GGTCAAGCTATTGTGGTTCATCCAGACTACTTATACGGTGCTATCACTGAAGACAGATAC
AACGAGTATGTCGCCAAGGTTAGTGCCAGAGAGAAAAGTGCCTACAAATTCTTCCATAAT
GGTATGATCTACAACAAGTTGTTCGTAAGTAAAGAGCATGCTCCATACACTGATGAATTG
GAAGAGGATGTTTACTTGGACCCATTAGCCCGTGTATCTAAGGATAAGAAATCAGGCTCC
TTGACTTTCAACTCTAAAAACATCCAAAGCAAGGACAGTTACATCAATGCTAACACCATT
GAAACTGCCAAGATGATTGAAAACATGACCAAGGAGAAAGTCTCTAACGGTGGCGTCGGT
GTAGATGTTGAATTAATCACTAGCATCAACGTTGAAAATGATACTTTTATCGAGCGCAAT
TTCACCCCGCAAGAAATAGAGTACTGCAGCGCGCAGCCTAGTGTGCAAAGCTCTTTCGCT
GGGACATGGTCCGCCAAAGAGGCTGTTTTCAAGTCCTTAGGCGTCAAGTCCTTAGGCGGT
GGTGCTGCATTGAAAGACATCGAAATCGTACGCGTTAACAAAAACGCTCCAGCCGTTGAA
CTGCACGGTAACGCCAAAAAGGCTGCCGAAGAAGCTGGTGTTACCGATGTGAAGGTATCT
ATTTCTCACGATGACCTCCAAGCTGTCGCGGTCGCCGTTTCTACTAAGAAATAG

>codon-optimized fusFAS, CAI change: 0.29 > 0.97
ATGGACGCTTACTCTACTAGACCATTGACTTTGTCTCACGGTTCTTTGGAACACGTTTTG
TTGGTTCCAACTGCTTCTTTCTTCATCGCTTCTCAATTGCAAGAACAATTCAACAAGATC
TTGCCAGAACCAACTGAAGGTTTCGCTGCTGACGACGAACCAACTACTCCAGCTGAATTG
GTTGGTAAGTTCTTGGGTTACGTTTCTTCTTTGGTTGAACCATCTAAGGTTGGTCAATTC
GACCAAGTTTTGAACTTGTGTTTGACTGAATTCGAAAACTGTTACTTGGAAGGTAACGAC
ATCCACGCTTTGGCTGCTAAGTTGTTGCAAGAAAACGACACTACTTTGGTTAAGACTAAG
GAATTGATCAAGAACTACATCACTGCTAGAATCATGGCTAAGAGACCATTCGACAAGAAG
TCTAACTCTGCTTTGTTCAGAGCTGTTGGTGAAGGTAACGCTCAATTGGTTGCTATCTTC
GGTGGTCAAGGTAACACTGACGACTACTTCGAAGAATTGAGAGACTTGTACCAAACTTAC
CACGTTTTGGTTGGTGACTTGATCAAGTTCTCTGCTGAAACTTTGTCTGAATTGATCAGA
ACTACTTTGGACGCTGAAAAGGTTTTCACTCAAGGTTTGAACATCTTGGAATGGTTGGAA
AACCCATCTAACACTCCAGACAAGGACTACTTGTTGTCTATCCCAATCTCTTGTCCATTG
ATCGGTGTTATCCAATTGGCTCACTACGTTGTTACTGCTAAGTTGTTGGGTTTCACTCCA
GGTGAATTGAGATCTTACTTGAAGGGTGCTACTGGTCACTCTCAAGGTTTGGTTACTGCT
GTTGCTATCGCTGAAACTGACTCTTGGGAATCTTTCTTCGTTTCTGTTAGAAAGGCTATC
ACTGTTTTGTTCTTCATCGGTGTTAGATGTTACGAAGCTTACCCAAACACTTCTTTGCCA
CCATCTATCTTGGAAGACTCTTTGGAAAACAACGAAGGTGTTCCATCTCCAATGTTGTCT
ATCTCTAACTTGACTCAAGAACAAGTTCAAGACTACGTTAACAAGACTAACTCTCACTTG
CCAGCTGGTAAGCAAGTTGAAATCTCTTTGGTTAACGGTGCTAAGAACTTGGTTGTTTCT
GGTCCACCACAATCTTTGTACGGTTTGAACTTGACTTTGAGAAAGGCTAAGGCTCCATCT

GGTTTGGACCAATCTAGAATCCCATTCTCTGAAAGAAAGTTGAAGTTCTCTAACAGATTC
TTGCCAGTTGCTTCTCCATTCCACTCTCACTTGTTGGTTCCAGCTTCTGACTTGATCAAC
AAGGACTTGGTTAAGAACAACGTTTCTTTCAACGCTAAGGACATCCAAATCCCAGTTTAC
GACACTTTCGACGGTTCTGACTTGAGAGTTTTGTCTGGTTCTATCTCTGAAAGAATCGTT
GACTGTATCATCAGATTGCCAGTTAAGTGGGAAACTACTACTCAATTCAAGGCTACTCAC
ATCTTGGACTTCGGTCCAGGTGGTGCTTCTGGTTTGGGTGTTTTGACTCACAGAAACAAG
GACGGTACTGGTGTTAGAGTTATCGTTGCTGGTACTTTGGACATCAACCCAGACGACGAC
TACGGTTTCAAGCAAGAAATCTTCGACGTTACTTCTAACGGTTTGAAGAAGAACCCAAAC
TGGTTGGAAGAATACCACCCAAAGTTGATCAAGAACAAGTCTGGTAAGATCTTCGTTGAA
ACTAAGTTCTCTAAGTTGATCGGTAGACCACCATTGTTGGTTCCAGGTATGACTCCATGT
ACTGTTTCTCCAGACTTCGTTGCTGCTACTACTAACGCTGGTTACACTATCGAATTGGCT
GGTGGTGGTTACTTCTCTGCTGCTGGTATGACTGCTGCTATCGACTCTGTTGTTTCTCAA
ATCGAAAAGGGTTCTACTTTCGGTATCAACTTGATCTACGTTAACCCATTCATGTTGCAA
TGGGGTATCCCATTGATCAAGGAATTGAGATCTAAGGGTTACCCAATCCAATTCTTGACT
ATCGGTGCTGGTGTTCCATCTTTGGAAGTTGCTTCTGAATACATCGAAACTTTGGGTTTG
AAGTACTTGGGTTTGAAGCCAGGTTCTATCGACGCTATCTCTCAAGTTATCAACATCGCT
AAGGCTCACCCAAACTTCCCAATCGCTTTGCAATGGACTGGTGGTAGAGGTGGTGGTCAC
CACTCTTTCGAAGACGCTCACACTCCAATGTTGCAAATGTACTCTAAGATCAGAAGACAC
CCAAACATCATGTTGATCTTCGGTTCTGGTTTCGGTTCTGCTGACGACACTTACCCATAC
TTGACTGGTGAATGGTCTACTAAGTTCGACTACCCACCAATGCCATTCGACGGTTTCTTG
TTCGGTTCTAGAGTTATGATCGCTAAGGAAGTTAAGACTTCTCCAGACGCTAAGAAGTGT
ATCGCTGCTTGTACTGGTGTTCCAGACGACAAGTGGGAACAAACTTACAAGAAGCCAACT
GGTGGTATCGTTACTGTTAGATCTGAAATGGGTGAACCAATCCACAAGATCGCTACTAGA
GGTGTTATGTTGTGGAAGGAATTCGACGAAACTATCTTCAACTTGCCAAAGAACAAGTTG
GTTCCAACTTTGGAAGCTAAGAGAGACTACATCATCTCTAGATTGAACGCTGACTTCCAA
AAGCCATGGTTCGCTACTGTTAACGGTCAAGCTAGAGACTTGGCTACTATGACTTACGAA
GAAGTTGCTAAGAGATTGGTTGAATTGATGTTCATCAGATCTACTAACTCTTGGTTCGAC
GTTACTTGGAGAACTTTCACTGGTGACTTCTTGAGAAGAGTTGAAGAAAGATTCACTAAG
TCTAAGACTTTGTCTTTGATCCAATCTTACTCTTTGTTGGACAAGCCAGACGAAGCTATC
GAAAAGGTTTTCAACGCTTACCCAGCTGCTAGAGAACAATTCTTGAACGCTCAAGACATC
GACCACTTCTTGTCTATGTGTCAAAACCCAATGCAAAAGCCAGTTCCATTCGTTCCAGTT
TTGGACAGAAGATTCGAAATCTTCTTCAAGAAGGACTCTTTGTGGCAATCTGAACACTTG
GAAGCTGTTGTTGACCAAGACGTTCAAAGAACTTGTATCTTGCACGGTCCAGTTGCTGCT
CAATTCACTAAGGTTATCGACGAACCAATCAAGTCTATCATGGACGGTATCCACGACGGT
CACATCAAGAAGTTGTTGCACCAATACTACGGTGACGACGAATCTAAGATCCCAGCTGTT
GAATACTTCGGTGGTGAATCTCCAGTTGACGTTCAATCTCAAGTTGACTCTTCTTCTGTT
TCTGAAGACTCTGCTGTTTTCAAGGCTACTTCTTCTACTGACGAAGAATCTTGGTTCAAG
GCTTTGGCTGGTTCTGAAATCAACTGGAGACACGCTTCTTTCTTGTGTTCTTTCATCACT
CAAGACAAGATGTTCGTTTCTAACCCAATCAGAAAGGTTTTCAAGCCATCTCAAGGTATG
GTTGTTGAAATCTCTAACGGTAACACTTCTTCTAAGACTGTTGTTACTTTGTCTGAACCA
GTTCAAGGTGAATTGAAGCCAACTGTTATCTTGAAGTTGTTGAAGGAAAACATCATCCAA
ATGGAAATGATCGAAAACAGAACTATGGACGGTAAGCCAGTTTCTTTGCCATTGTTGTAC
AACTTCAACCCAGACAACGGTTTCGCTCCAATCTCTGAAGTTATGGAAGACAGAAACCAA
AGAATCAAGGAAATGTACTGGAAGTTGTGGATCGACGAACCATTCAACTTGGACTTCGAC
CCAAGAGACGTTATCAAGGGTAAGGACTTCGAAATCACTGCTAAGGAAGTTTACGACTTC
ACTCACGCTGTTGGTAACAACTGTGAAGACTTCGTTTCTAGACCAGACAGAACTATGTTG
GCTCCAATGGACTTCGCTATCGTTGTTGGTTGGAGAGCTATCATCAAGGCTATCTTCCCA
AACACTGTTGACGGTGACTTGTTGAAGTTGGTTCACTTGTCTAACGGTTACAAGATGATC
CCAGGTGCTAAGCCATTGCAGGTTGGTGACGTTGTTTCTACTACTGCTGTTATCGAATCT
GTTGTTAACCAACCAACTGGTAAGATCGTTGACGTTGTTGGTACTTTGTCTAGAAACGGT
AAGCCAGTTATGGAAGTTACTTCTTCTTTCTTCTACAGAGGTAACTACACTGACTTCGAA
AACACTTTCCAAAAGACTGTTGAACCAGTTTACCAAATGCACATCAAGACTTCTAAGGAC
ATCGCTGTTTTGAGATCTAAGGAATGGTTCCAATTGGACGACGAAGACTTCGACTTGTTG
AACAAGACTTTGACTTTCGAAACTGAAACTGAAGTTACTTTCAAGAACGCTAACATCTTC
TCTTCTGTTAAGTGTTTCGGTCCAATCAAGGTTGAATTGCCAACTAAGGAAACTGTTGAA
ATCGGTATCGTTGACTACGAAGCTGGTGCTTCTCACGGTAACCCAGTTGTTGACTTCTTG
AAGAGAAACGGTTCTACTTTGGAACAAAAGGTTAACTTGGAAAACCCAATCCCAATCGCT
GTTTTGGACTCTTACACTCCATCTACTAACGAACCATACGCTAGAGTTTCTGGTGACTTG
AACCCAATCCACGTTTCTAGACACTTCGCTTCTTACGCTAACTTGCCAGGTACTATCACT
CACGGTATGTTCTCTTCTGCTTCTGTTAGAGCTTTGATCGAAAACTGGGCTGCTGACTCT
GTTTCTTCTAGAGTTAGAGGTTACACTTGTCAATTCGTTGACATGGTTTTGCCAAACACT
GCTTTGAAGACTTCTATCCAACACGTTGGTATGATCAACGGTAGAAAGTTGATCAAGTTC

GAAACTAGAAACGAAGACGACGTTGTTGTTTTGACTGGTGAAGCTGAAATCGAACAACCA
GTTACTACTTTCGTTTTCACTGGTCAAGGTTCTCAAGAACAAGGTATGGGTATGGACTTG
TACAAGACTTCTAAGGCTGCTCAAGACGTTTGGAACAGAGCTGACAACCACTTCAAGGAC
ACTTACGGTTTCTCTATCTTGGACATCGTTATCAACAACCCAGTTAACTTGACTATCCAC
TTCGGTGGTGAAAAGGGTAAGAGAATCAGAGAAAACTACTCTGCTATGATCTTCGAAACT
ATCGTTGACGGTAAGTTGAAGACTGAAAAGATCTTCAAGGAAATCAACGAACACTCTACT
TCTTACACTTTCAGATCTGAAAAGGGTTTGTTGTCTGCTACTCAATTCACTCAACCAGCT
TTGACTTTGATGGAAAAGGCTGCTTTCGAAGACTTGAAGTCTAAGGGTTTGATCCCAGCT
GACGCTACTTTCGCTGGTCACTCTTTGGGTGAATACGCTGCTTTGGCTTCTTTGGCTGAC
GTTATGTCTATCGAATCTTTGGTTGAAGTTGTTTTCTACAGAGGTATGACTATGCAAGTT
GCTGTTCCAAGAGACGAATTGGGTAGATCTAACTACGGTATGATCGCTATCAACCCAGGT
AGAGTTGCTGCTTCTTTCTCTCAAGAAGCTTTGCAATACGTTGTTGAAAGAGTTGGTAAG
AGAACTGGTTGGTTGGTTGAAATCGTTAACTACAACGTTGAAAACCAACAATACGTTGCT
GCTGGTGACTTGAGAGCTTTGGACACTGTTACTAACGTTTTGAACTTCATCAAGTTGCAA
AAGATCGACATCATCGAATTGCAAAAGTCTTTGTCTTTGGAAGAAGTTGAAGGTCACTTG
TTCGAAATCATCGACGAAGCTTCTAAGAAGTCTGCTGTTAAGCCAAGACCATTGAAGTTG
GAAAGAGGTTTCGCTTGTATCCCATTGGTTGGTATCTCTGTTCCATTCCACTCTACTTAC
TTGATGAACGGTGTTAAGCCATTCAAGTCTTTCTTGAAGAAGAACATCATCAAGGAAAAC
GTTAAGGTTGCTAGATTGGCTGGTAAGTACATCCCAAACTTGACTGCTAAGCCATTCCAA
GTTACTAAGGAATACTTCCAAGACGTTTACGACTTGACTGGTTCTGAACCAATCAAGGAA
ATCATCGACAACTGGGAAAAGGACGAATGGGGTTCTCCAGAAGTTGAACAAGAATTGGCT
CACATCTTGTTGACTGAATTGTTGGCTTACCAATTCGCTTCTCCAGTTAGATGGATCGAA
ACTCAAGACGTTTTCTTGAAGGACTTCAACACTGAAAGAGTTGTTGAAATCGGTCCATCT
CCAACTTTGGCTGGTATGGCTCAAAGAACTTTGAAGAACAAGTACGAATCTTACGACGCT
GCTTTGTCTTTGCACAGAGAAATCTTGTGTTACTCTAAGGACGCTAAGGAAATCTACTAC
ACTCCAGACCCATCTGAATTGGCTGCTAAGGAAGAACCAGCTAAGGAAGAAGCTCCAGCT
CCAACTCCAGCTGCTTCTGCTCCAGCTCCAGCTGCCGCTGCCCCAGCTCCAGTTGCTGCC
GCCGCTCCAGCTGCTGCTGCTGCTGAAATCGCTGACGAACCAGTTAAGGCTTCTTTGTTG
TTGCACGTTTTGGTTGCTCACAAGTTGAAGAAGTCTTTGGACTCTATCCCAATGTCTAAG
ACTATCAAGGACTTGGTTGGTGGTAAGTCTACTGTTCAAAACGAAATCTTGGGTGACTTG
GGTAAGGAATTCGGTACTACTCCAGAAAAGCCAGAAGAAACTCCATTGGAAGAATTGGCT
GAAACTTTCCAAGACACTTTCTCTGGTGCTTTGGGTAAGCAATCTTCTTCTTTGTTGTCT
AGATTGATCTCTTCTAAGATGCCAGGTGGTTTCACTATCACTGTTGCTAGAAAGTACTTG
CAAACTAGATGGGGTTTGCCATCTGGTAGACAAGACGGTGTTTTGTTGGTTGCTTTGTCT
AACGAACCAGCTGCTAGATTGGGTTCTGAAGCTGACGCTAAGGCTTTCTTGGACTCTATG
GCTCAAAAGTACGCTTCTATCGTTGGTGTTGACTTGTCTTCTGCTGCTTCTGCTTCTGGA
GCTGCTGGTGCTGGTGCTGCTGCTGGCGCTGCTATGATCGACGCTGGTGCTTTGGAAGAA
ATCACTAAGGACCACAAGGTTTTGGCTAGACAACAATTGCAAGTTTTGGCTAGATACTTG
AAGATGGACTTGGACAACGGTGAAAGAAAGTTCTTGAAGGAAAAGGACACTGTTGCTGAA
TTGCAAGCTCAATTGGACTACTTGAACGCTGAATTGGGTGAATTCTTCGTTAACGGTGTT
GCTACTTCTTTCAGTAGAAAGAAGGCTAGAACTTTCGACTCTTCTTGGAACTGGGCTAAG
CAATCTTTGTTGTCTTTGTACTTCGAAATCATCCACGGTGTTTTGAAGAACGTTGACAGA
GAAGTTGTTTCTGAAGCTATCAACATCATGAACAGATCTAACGACGCTTTGATCAAGTTC
ATGGAATACCACATCTCTAACACTGACGAAACTAAGGGTGAAAACTACCAATTGGTTAAG
ACTTTGGGTGAACAATTGATCGAAAACTGTAAGCAAGTTTTGGACGTTGACCCAGTTTAC
AAGGACGTTGCTAAGCCAACTGGTCCAAAGACTGCTATCGACAAGAACGGTAACATCACT
TACTCTGAAGAACCAAGAGAAAAGGTTAGAAAGTTGTCTCAATACGTTCAAGAAATGGCT
TTGGGTGGTCCAATCACTAAGGAATCTCAACCAACTATCGAAGAAGACTTGACTAGAGTT
TACAAGGCTATCTCTGCTCAAGCTGACAAGCAAGACATCTCTTCTTCTACTAGAGTTGAA
TTCGAAAAGTTGTACTCTGACTTGATGAAGTTCTTGGAATCTTCTAAGGAAATCGACCCA
TCTCAAACTACTCAATTGGCTGGTATGGACGTTGAAGACGCTTTGGACAAGGACTCTACT
AAGGAAGTTGCTTCTTTGCCAAACAAGTCTACTATCTCTAAGACTGTTTCTTCTACTATC
CCAAGAGAAACTATCCCATTCTTGCACTTGAGAAAGAAGACTCCAGCTGGTGACTGGAAG
TACGACAGACAATTGTCTTCTTTGTTCTTGGACGGTTTGGAAAAGGCTGCTTTCAACGGT
GTTACTTTCAAGGACAAGTACGTTTTGATCACTGGTGCTGGTAAGGGTTCTATCGGTGCT
GAAGTTTTGCAAGGTTTGTTGCAAGGTGGTGCTAAGGTTGTTGTTACTACTTCTAGATTC
TCTAAGCAAGTTACTGACTACTACCAATCTATCTACGCTAAGTACGGTGCTAAGGGTTCT
ACTTTGATCGTTGTTCCATTCAACCAAGGTTCTAAGCAAGACGTTGAAGCTTTGATCGAA
TTCATCTACGACACTGAAAAGAACGGTGGTTTGGGTTGGGACTTGGACGCTATCATCCCA
TTCGCTGCTATCCCAGAACAAGGTATCGAATTGGAACACATCGACTCTAAGTCTGAATTC
GCTCACAGAATCATGTTGACTAACATCTTGAGAATGATGGGTTGTGTTAAGAAGCAAAAG
TCTGCTAGAGGTATCGAAACTAGACCAGCTCAAGTTATCTTGCCAATGTCTCCAAACCAC

GGTACTTTCGGTGGTGACGGTATGTACTCTGAATCTAAGTTGTCTTTGGAAACTTTGTTC
AACAGATGGCACTCTGAATCTTGGGCTAACCAATTGACTGTTTGTGGTGCTATCATCGGT
TGGACTAGAGGTACTGGTTTGATGTCTGCTAACAACATCATCGCTGAAGGTATCGAAAAG
ATGGGTGTTAGAACTTTCTCTCAAAAGGAAATGGCTTTCAACTTGTTGGGTTTGTTGACT
CCAGAAGTTGTTGAATTGTGTCAAAAGTCTCCAGTTATGGCTGACTTGAACGGTGGTTTG
CAATTCGTTCCAGAATTGAAGGAATTCACTGCTAAGTTGAGAAAGGAATTGGTTGAAACT
TCTGAAGTTAGAAAGGCTGTTTCTATCGAAACTGCTTTGGAACACAAGGTTGTTAACGGT
AACTCTGCTGACGCTGCTTACGCTCAAGTTGAAATCCAACCAAGAGCTAACATCCAATTG
GACTTCCCAGAATTGAAGCCATACAAGCAAGTTAAGCAAATCGCTCCAGCTGAATTGGAA
GGTTTGTTGGACTTGGAAAGAGTTATCGTTGTTACTGGTTTCGCTGAAGTTGGTCCATGG
GGTTCTGCTAGAACTAGATGGGAAATGGAAGCTTTCGGTGAATTCTCTTTGGAAGGTTGT
GTTGAAATGGCTTGGATCATGGGTTTCATCTCTTACCACAACGGTAACTTGAAGGGTAGA
CCATACACTGGTTGGGTTGACTCTAAGACTAAGGAACCAGTTGACGACAAGGACGTTAAG
GCTAAGTACGAAACTTCTATCTTGGAACACTCTGGTATCAGATTGATCGAACCAGAATTG
TTCAACGGTTACAACCCAGAAAAGAAGGAAATGATCCAAGAAGTTATCGTTGAAGAAGAC
TTGGAACCATTCGAAGCTTCTAAGGAAACTGCTGAACAATTCAAGCACCAACACGGTGAC
AAGGTTGACATCTTCGAAATCCCAGAAACTGGTGAATACTCTGTTAAGTTGTTGAAGGGT
GCTACTTTGTACATCCCAAAGGCTTTGAGATTCGACAGATTGGTTGCTGGTCAAATCCCA
ACTGGTTGGAACGCTAAGACTTACGGTATCTCTGACGACATCATCTCTCAAGTTGACCCA
ATCACTTTGTTCGTTTTGGTTTCTGTTGTTGAAGCTTTCATCGCTTCTGGTATCACTGAC
CCATACGAAATGTACAAGTACGTTCACGTTTCTGAAGTTGGTAACTGTTCTGGTTCTGGT
ATGGGTGGTGTTTCGGCTTTGAGAGGTATGTTCAAGGACAGATTCAAGGACGAACCAGTT
CAAAACGACATCTTGCAAGAATCTTTCATCAACACTATGTCTGCTTGGGTTAACATGTTG
TTGATCTCTTCTTCTGGTCCAATCAAGACTCCAGTTGGTGCTTGTGCTACTTCTGTTGAA
TCTGTTGACATCGGTGTTGAAACTATCTTGTCTGGTAAGGCTAGAATCTGTATCGTTGGT
GGTTACGACGACTTCCAAGAAGAAGGTTCTTTCGAATTCGGTAACATGAAGGCTACTTCT
AACACTTTGGAAGAATTCGAACACGGTAGAACTCCAGCTGAAATGTCTAGACCAGCTACT
ACTACTAGAAACGGTTTCATGGAAGCTCAAGGTGCTGGTATCCAAATCATCATGCAAGCT
GACTTGGCTTTGAAGATGGGTGTTCCAATCTACGGTATCGTTGCTATGGCTGCTACTGCT
ACTGACAAGATCGGTAGATCTGTTCCAGCTCCAGGTAAGGGTATCTTGACTACTGCTAGA
GAACACCACTCTTCTGTTAAGTACGCTTCTCCAAACTTGAACATGAAGTACAGAAAGAGA
CAATTGGTTACTAGAGAAGCTCAAATCAAGGACTGGGTTGAAAACGAATTGGAAGCTTTG
AAGTTGGAAGCTGAAGAAATCCCATCTGAAGACCAAAACGAATTCTTGTTGGAAAGAACT
AGAGAAATCCACAACGAAGCTGAATCTCAATTGAGAGCTGCTCAACAACAATGGGGTAAC
GACTTCTACAAGAGAGACCCAAGAATCGCTCCATTGAGAGGTGCTTTGGCTACTTACGGT
TTGACTATCGACGACTTGGGTGTTGCTTCTTTCCACGGTACTTCTACTAAGGCTAACGAC
AAGAACGAATCTGCTACTATCAACGAAATGATGAAGCACTTGGGTAGATCTGAAGGTAAC
CCAGTTATCGGTGTTTTCCAAAAGTTCTTGACTGGTCACCCAAAGGGTGCTGCTGGTGCT
TGGATGATGAACGGTGCTTTGCAAATCTTGAACTCTGGTATCATCCCAGGTAACAGAAAC
GCTGACAACGTTGACAAGATCTTGGAACAATTCGAATACGTTTTGTACCCATCTAAGACT
TTGAAGACTGACGGTGTTAGAGCTGTTTCTATCACTTCTTTCGGTTTCGGTCAAAAGGGT
GGTCAAGCTATCGTTGTTCACCCAGACTACTTGTACGGTGCTATCACTGAAGACAGATAC
AACGAATACGTTGCTAAGGTTTCTGCTAGAGAAAAGTCTGCTTACAAGTTCTTCCACAAC
GGTATGATCTACAACAAGTTGTTCGTTTCTAAGGAACACGCTCCATACACTGACGAATTG
GAAGAAGACGTTTACTTGGACCCATTGGCTAGAGTTTCTAAGGACAAGAAGTCTGGTTCT
TTGACTTTCAACTCTAAGAACATCCAATCTAAGGACTCTTACATCAACGCTAACACTATC
GAAACTGCTAAGATGATCGAAAACATGACTAAGGAAAAGGTTTCTAACGGTGGTGTTGGT
GTTGACGTTGAATTGATCACTTCTATCAACGTTGAAAACGACACTTTCATCGAAAGAAAC
TTCACTCCACAAGAAATCGAATACTGTTCTGCTCAACCATCTGTTCAATCTTCTTTCGCT
GGTACTTGGTCTGCTAAGGAAGCTGTTTTCAAGTCTTTGGGTGTTAAGTCTTTGGGTGGT
GGTGCTGCTTTGAAGGACATCGAAATCGTTAGAGTTAACAAGAACGCTCCAGCTGTTGAA
TTGCACGGTAACGCTAAGAAGGCTGCTGAAGAAGCTGGTGTTACTGACGTTAAGGTTTCT
ATCTCTCACGACGACTTGCAAGCTGTTGCTGTTGCTGTTTCTACTAAGAAGTAA

Supplementary Information - References

Henritzi, S., Fischer, M., Grininger, M., Oreb, M., and Boles, E. (2018). An engineered fatty acid synthase combined with a carboxylic acid reductase enables de novo production of 1-octanol in Saccharomyces cerevisiae. *Biotechnol. Biofuels* 11, 150. doi:10.1016/j.jtrangeo.2006.11.008

Farwick A, Bruder S, Schadeweg V, Oreb M, Boles E (2014) Engineering of yeast hexose transporters to transport D-xylose without inhibition by D-glucose. *Proc. Natl. Acad. Sci. USA* 111: 5159–5164. doi:10.1073/pnas.1323464111
